# Heterozygous *BTNL8* variants in individuals with multisystem inflammatory syndrome in children (MIS-C)

**DOI:** 10.1084/jem.20240699

**Published:** 2024-11-22

**Authors:** Evangelos Bellos, Dilys Santillo, Pierre Vantourout, Heather R. Jackson, Amedine Duret, Henry Hearn, Yoann Seeleuthner, Estelle Talouarn, Stephanie Hodeib, Harsita Patel, Oliver Powell, Sophya Yeoh, Sobia Mustafa, Dominic Habgood-Coote, Samuel Nichols, Leire Estramiana Elorrieta, Giselle D’Souza, Victoria J. Wright, Diego Estrada-Rivadeneyra, Adriana H. Tremoulet, Kirsten B. Dummer, Stejara A. Netea, Antonio Condino-Neto, Yu Lung Lau, Esmeralda Núñez Cuadros, Julie Toubiana, Marisol Holanda Pena, Frédéric Rieux-Laucat, Charles-Edouard Luyt, Filomeen Haerynck, Jean Louis Mège, Samya Chakravorty, Elie Haddad, Marie-Paule Morin, Özge Metin Akcan, Sevgi Keles, Melike Emiroglu, Gulsum Alkan, Sadiye Kübra Tüter Öz, Sefika Elmas Bozdemir, Guillaume Morelle, Alla Volokha, Yasemin Kendir-Demirkol, Betul Sözeri, Taner Coskuner, Aysun Yahsi, Belgin Gulhan, Saliha Kanik-Yuksek, Gulsum Iclal Bayhan, Aslinur Ozkaya-Parlakay, Osman Yesilbas, Nevin Hatipoglu, Tayfun Ozcelik, Alexandre Belot, Emilie Chopin, Vincent Barlogis, Esra Sevketoglu, Emin Menentoglu, Zeynep Gokce Gayretli Aydin, Marketa Bloomfield, Suzan A. AlKhater, Cyril Cyrus, Yuriy Stepanovskiy, Anastasiia Bondarenko, Fatma Nur Öz, Meltem Polat, Jiří Fremuth, Jan Lebl, Amyrath Geraldo, Emmanuelle Jouanguy, COVID-19 Human Genetic Effort, Alessandro Aiuti, Laia Alsina, Evangelos Andreakos, Ana de Andrés Martín, Catherine M. Biggs, Alessandro Borghesi, Amed Aziz Bousfiha, Daniel Blazquez-Gamero, Petter Brodin, Giorgio Casari, Antonio Condino-Neto, Jacques Fellay, Carlos Flores, Guy Gorochov, Emine Hafize Erdeniz, Lennart Hammarström, Yolanda Jordan Garcia, Adem Karbuz, Adam Klocperk, Paloma Lapuente-Suanzes, Yu-Lung Lau, Cheng Leung, Davood Mansouri, Trine H. Mogensen, Lisa F.P. Ng, Antonio Novelli, Giuseppe Novelli, Keisuke Okamoto, Satoshi Okada, Qiang Pan-Hammarström, Graziano Pesole, Laurent Renia, Carlos Rodriguez-Gallego, Anna Sediva, Mohammad Shahrooei, Pere Soler-Palacin, András N. Spaan, Gonul Tanir, Ahmad Abou Tayoun, Şehime G. Temel, Donald C. Vinh, DIAMONDS, Aubrey Cunnington, Jethro Herberg, Myrsini Kaforou, Victoria J. Wright, Evangelos Bellos, Claire Broderick, Samuel Channon-Wells, Samantha Cooray, Tisham De, Giselle D'Souza, Leire Estramia Elorrieta, Diego Estrada-Rivadeneyra, Rachel Galassini, Dominic Habgood-Coote, Shea Hamilton, Heather Jackson, James Kavangh, Ila Keren, Mahdi Moradi Marjaneh, Stephanie Menikou, Samuel Nichols, Ruud Nijman, Ivana Pennisi, Oliver Powell, Ruth Reid, Priyen Shah, Ortensia Vito, Elizabeth Whittaker, Clare Wilson, Rebecca Womersley, Ami Abdulla, Sarah Darnell, Sobia Mustafa, Pantelis Georgiou, Jesus-Rodriguez Manzano, Nicolas Moser, Michael Carter, Shane Tibby, Jonathan Cohen, Francesca Davis, Julia Kenny, Paul Wellman, Marie White, Matthew Fish, Aislinn Jennings, Manu Shankar-Hari, Katy Fidler, Dan Agranoff, Vivien Richmond, Mathhew Seal, Saul Faust, Dan Owen, Ruth Ensom, Sarah McKay, Mariya Shaji, Rachel Schranz, Prita Rughni, Amutha Anpanthar, Susan Liebeschuetz, Anna Riddell, Divya Divakaran, Louise Han, Nosheen Khalid, Ivone Lancoma Malcolm, Jessica Schofield, Teresa Simagan, Mark Peters, Alasdair Bamford, Lauran O'Neill, Nazima Pathan, Esther Daubney, Deborah White, Melissa Heightman, Sarah Eisen, Terry Segal, Lucy Wellings, Simon B. Drysdale, Nicole Branch, Lisa Hamzah, Heather Jarman, Maggie Nyirenda, Lisa Capozzi, Emma Gardiner, Robert Moots, Magda Nasher, Anita Hanson, Michelle Linforth, Sean O'Riordan, Don Ellis, Akash Deep, Ivan Caro, Fiona Shackley, Arianna Bellini, Stuart Gormley, Samira Neshat, Barnaby Scholefield, Ceri Robbins, Helen Winmill, Stéphane C. Paulus, Andrew J. Pollard, Mark Anthony, Sarah Hopton, Danielle Miller, Zoe Oliver, Sally Beer, Bryony Ward, Shrija Shrestha, Meeru Gurung, Puja Amatya, Bhishma Pokhrel, Sanjeev Man Bijukchhe, Madhav Chandra Gautam, Sarah Kelly, Peter O'Reilly, Sonu Shrestha, Federico Martinón-Torres, Antonio Salas, Fernando Álvez González, Sonia Ares Gómez, Xabier Bello, Mirian Ben García, Fernando Caamaño Viña, Sandra Carnota, María José Curras-Tuala, Ana Dacosta Urbieta, Carlos Durán Suárez, Isabel Ferreiros Vidal, Luisa García Vicente, Alberto Gómez-Carballa, Jose Gómez Rial, Pilar Leboráns Iglesias, Narmeen Mallah, Nazareth Martinón-Torres, José María Martinón, Belén Mosquera Pérez Sánchez, Jacobo Pardo-Seco, Sara Pischedda, Sara Rey Vázquez, Irene Rivero Calle, Carmen Rodríguez-Tenreiro, Lorenzo Redondo-Collazo, Sonia Serén Fernández, Marisol Vilas Iglesias, Enitan D. Carrol, Elizabeth Cocklin, Rebecca Beckley, Abbey Bracken, Ceri Evans, Aakash Khanijau, Rebecca Lenihan, Nadia Lewis-Burke, Karen Newall, Sam Romaine, Jennifer Whitbread, Maria Tsolia, Irini Eleftheriou, Nikos Spyridis, Maria Tambouratzi, Despoina Maritsi, Antonios Marmarinos, Marietta Xagorari, Lourida Panagiota, Pefanis Aggelos, Akinosoglou Karoli, Gogos Charalambos, Maragos Markos, Voulgarelis Michalis, Stergiou Ioan, Marieke Emonts, Emma Lim, John Isaacs, Kathryn Bell, Stephen Crulley, Daniel Fabian, Evelyn Thomson, Diane Wallia, Caroline Miller, Ashley Bell, Fabian J.S. van der Velden, Geoff Shenton, Ashley Price, Owen Treloar, Daisy Thomas, Pablo Rojo, Cristina Epalza, Serena Villaverde, Sonia Márquez, Manuel Gijón, Fátima Machín, Laura Cabello, Irene Hernández, Lourdes Gutiérrez, Ángela Manzanares, Taco W. Kuijpers, Martijn van de Kuip, Marceline van Furth, Merlijn van den Berg, Giske Biesbroek, Floris Verkuil, Carlijn van der Zee, Dasja Pajkrt, Michael Boele van Hensbroek, Dieneke Schonenberg, Mariken Gruppen, Sietse Nagelkerke, Machiel H. Jansen, Ines Goetschalckx, Lorenza Romani, Maia De Luca, Sara Chiurchiù, Costanza Tripiciano, Stefania Mercadante, Clementien L. Vermont, Henriëtte A. Moll, Dorine M. Borensztajn, Nienke N. Hagedoorn, Chantal Tan, Joany Zachariasse, W. Dik, Ching-Fen Shen, Dace Zavadska, Sniedze Laivacuma, Aleksandra Rudzate, Diana Stoldere, Arta Barzdi, Elza Barzdi, Monta Madelane, Dagne Gravele, Dace Svilz, Romain Basmaci, Noémie Lachaume, Pauline Bories, RajaBen Tkhayat, Laura Chériaux, Juraté Davoust, Kim-Thanh Ong, Marie Cotillon, Thibault de Groc, Sébastien Le, Nathalie Vergnault, Hélène Sée, Laure Cohen, Alice de Tugny, Nevena Danekova, Marine Mommert-Tripon, Karen Brengel-Pesce, Marko Pokorn, Mojca Kolnik, Tadej Avčin, Tanja Avramoska, Natalija Bahovec, Petra Bogovič, Lidija Kitanovski, Mirijam Nahtigal, Lea Papst, Tina Plankar Srovin, Franc Strle, Katarina Vincek, Michiel van der Flier, Wim J.E. Tissing, Roelie M. Wösten-van Asperen, Sebastiaan J. Vastert, Daniel C. Vijlbrief, Louis J. Bont, Coco R. Beudeker, Philipp Agyeman, Luregn Schlapbach, Christoph Aebi, Mariama Usman, Stefanie Schlüchter, Verena Wyss, Nina Schöbi, Elisa Zimmermann, Marion Meier, Kathrin Weber, Colin Fink, Marie Voice, Leo Calvo-Bado, Michael Steele, Jennifer Holden, Andrew Taylor, Ronan Calvez, Catherine Davies, Benjamin Evans, Jake Stevens, Peter Matthews, Kyle Billing, Werner Zenz, Alexander Binder, Benno Kohlmaier, Daniela S. Kohlfürst, Nina A. Schweintzger, Christoph Zurl, Susanne Hösele, Manuel Leitner, Lena Pölz, Alexandra Rusu, Glorija Rajic, Bianca Stoiser, Martina Strempfl, Manfred G. Sagmeister, Sebastian Bauchinger, Martin Benesch, Astrid Ceolotto, Ernst Eber, Siegfried Gallistl, Harald Haidl, Almuthe Hauer, Christa Hude, Andreas Kapper, Markus Keldorfer, Sabine Löffler, Tobias Niedrist, Heidemarie Pilch, Andreas Pfleger, Klaus Pfurtscheller, Siegfried Rödl, Andrea Skrabl-Baumgartner, Volker Strenger, Elmar Wallner, Maike K. Tauchert, Shunmay Yeung, Manuel Dewez, David Bath, Elizabeth Fitchett, Fiona Cresswell, Effua Usuf, Kalifa Bojang, Anna Roca, Isatou Sarr, Momodou Saidykhan, Ebrahim Ndure, Ulrich von Both, Laura Kolberg, Patricia Schmied, Ioanna Mavridi, Irene Alba-Alejandre, Nikolaus Haas, Esther Maier, Sabrina Juranek, Tobias Feuchtinger, Katharina Danhauser, Matthias Griese, Matthias Kappler, Eberhard Lurz, Sebastian Schroepf, Florian Hoffmann, Karl Reiter, Carola Schoen, Luregn J. Schlapbach, Eric Giannoni, Martin Stocker, Klara M. Posfay-Barbe, Ulrich Heininger, Sara Bernhard-Stirnemann, Anita Niederer-Loher, Christian Kahlert, Giancarlo Talucci, Christa Relly, Christoph Berger, Thomas Riedel, Pedro Madrigal, Silvie Fexova, EUCLIDS, Michael Levin, Lachlan Coin, Stuart Gormley, Shea Hamilton, Jethro Herberg, Bernardo Hourmat, Clive Hoggart, Myrsini Kaforou, Vanessa Sancho-Shimizu, Victoria Wright, Amina Abdulla, Paul Agapow, Maeve Bartlett, Evangelos Bellos, Hariklia Eleftherohorinou, Rachel Galassini, David Inwald, Meg Mashbat, Stephanie Menikou, Sobia Mustafa, Simon Nadel, Rahmeen Rahman, Hannah Shailes, Clare Thakker, Sumit Bokhandi, Sue Power, Heather Barham, Nazima Pathan, Jenna Ridout, Deborah White, Sarah Thurston, Saul Faust, Sanjay Patel, Jenni McCorkell, Patrick Davies, Lindsey Crate, Helen Navarra, Stephanie Carter, Raghu Ramaiah, Rekha Patel, Catherine Tuffrey, Andrew Gribbin, Sharon McCready, Mark Peters, Katie Hardy, Fran Standing, Lauren O'Neill, Eugenia Abelake, Akash Deep, Eniola Nsirim, Andrew Pollard, Louise Willis, Zoe Young, Collin Royad, Sonia White, Peter-Marc Fortune, Phil Hudnott, Federico Martinón-Torres, Antonio Salas, Fernando Álvez González, Ruth Barral-Arca, Miriam Cebey-López, María José Curras-Tuala, Natalia García, Luisa García Vicente, Alberto Gómez-Carballa, Jose Gómez Rial, Andrea Grela Beiroa, Antonio Justicia Grande, Pilar Leboráns Iglesias, Alba Elena Martínez Santos, Federico Martinón-Torres, Nazareth Martinón-Torres, José María Martinón Sánchez, Beatriz Morillo Gutiérrez, Belén Mosquera Pérez, Pablo Obando Pacheco, Jacobo Pardo-Seco, Sara Pischedda, Irene Rivero-Calle, Carmen Rodríguez-Tenreiro, Lorenzo Redondo-Collazo, Sonia Serén Fernández, María del Sol Porto Silva, Ana Vega, Lucía Vilanova Trillo, Susana Beatriz Reyes, María Cruz León León, Álvaro Navarro Mingorance, Xavier Gabaldó Barrios, Eider Oñate Vergara, Andrés Concha Torre, Ana Vivanco, Reyes Fernández, Francisco Giménez Sánchez, Miguel Sánchez Forte, Pablo Rojo, Jesus Ruiz Contreras, Alba Palacios, Cristina Epalza Ibarrondo, Elizabeth Fernández Cooke, Marisa Navarro, Cristina Álvarez Álvarez, María José Lozano, Eduardo Carreras, Sonia Brió Sanagustín, Olaf Neth, Maria del Carmen Martínez Padilla, Luis Manuel Prieto Tato, Sara Guillén, Laura Fernández Silveira, David Moreno, Ronald de Groot, A. Marceline Tutu van Furth, Michiel van der Flier, Navin P. Boeddha, Gertjan J.A. Driessen, Jan Hazelzet, Taco W. Kuijpers, Dasja Pajkrt, Elisabeth A.M. Sanders, Diederik van de Beek, Arie van der Ende, Ria H.L.A. Philipsen, Abdul O.A. Adeel, Mijke A. Breukels, Danielle M.C. Brinkman, Carla C.M.M. de Korte, Esther de Vries, Wouter J. de Waal, Roel Dekkers, Anouk Dings-Lammertink, Rienus A. Doedens, Albertine E. Donker, Mieke Dousma, Tina E. Faber, Gerardus P.J.M. Gerrits, Jan A.M. Gerver, Jojanneke Heidema, Jenneke Homan-van der Veen, Monique A.M. Jacobs, Nicolaas J.G. Jansen, Pawel Kawczynski, Kristine Klucovska, Martin C.J. Kneyber, Yvonne Koopman-Keemink, Veerle J. Langenhorst, José Leusink, Bettina F. Loza, Istvan T. Merth, Carien J. Miedema, Chris Neeleman, Jeroen G. Noordzij, Charles C. Obihara, A. Lindy T. van Overbeek - van Gils, Geriska H. Poortman, Stephanus T. Potgieter, Joke Potjewijd, Phillippe P.R. Rosias, Tom Sprong, Gavin W. ten Tussher, Boony J. Thio, Gerdien A. Tramper-Stranders, Marcel van Deuren, Henny van der Meer, Andre J.M. van Kuppevelt, Anne-Marie van Wermeskerken, Wim A. Verwijs, Tom F.W. Wolfs, Luregn J. Schlapbach, Philipp Agyeman, Christoph Aebi, Christoph Berger, Eric Giannoni, Martin Stocker, Klara M. Posfay-Barbe, Ulrich Heininger, Sara Bernhard-Stirnemann, Anita Niederer-Loher, Christian Kahlert, Paul Hasters, Christa Relly, Walter Baer, Enitan D. Carrol, Stéphane Paulus, Hannah Frederick, Rebecca Jennings, Joanne Johnston, Rhian Kenwright, Colin G. Fink, Elli Pinnock, Marieke Emonts, Rachel Agbeko, Suzanne Anderson, Fatou Secka, Kalifa Bojang, Isatou Sarr, Ngange Kebbeh, Gibbi Sey, Momodou Saidykhan, Fatoumata Cole, Gilleh Thomas, Martin Antonio, Werner Zenz, Daniela S. Kohlfürst, Alexander Binder, Nina A. Schweintzger, Manfred Sagmeister, Hinrich Baumgart, Markus Baumgartner, Uta Behrends, Ariane Biebl, Robert Birnbacher, Jan-Gerd Blanke, Carsten Boelke, Kai Breuling, Jürgen Brunner, Maria Buller, Peter Dahlem, Beate Dietrich, Ernst Eber, Johannes Elias, Josef Emhofer, Rosa Etschmaier, Sebastian Farr, Ylenia Girtler, Irina Grigorow, Konrad Heimann, Ulrike Ihm, Zdenek Jaros, Hermann Kalhoff, Wilhelm Kaulfersch, Christoph Kemen, Nina Klocker, Bernhard Köster, Benno Kohlmaier, Eleni Komini, Lydia Kramer, Antje Neubert, Daniel Ortner, Lydia Pescollderungg, Klaus Pfurtscheller, Karl Reiter, Goran Ristic, Siegfried Rödl, Andrea Sellner, Astrid Sonnleitner, Matthias Sperl, Wolfgang Stelzl, Holger Till, Andreas Trobisch, Anne Vierzig, Ulrich Vogel, Christina Weingarten, Stefanie Welke, Andreas Wimmer, Uwe Wintergerst, Daniel Wüller, Andrew Zaunschirm, Ieva Ziuraite, Veslava Žukovskaja, Martin L. Hibberd, Sonia Davila, Isabel Delany, Michael J. Carter, Paul Wellman, Mark Peters, Rebeca Pérez de Diego, Lindsey Ann Edwards, Christopher Chiu, Mahdad Noursadeghi, Alexandre Bolze, Chisato Shimizu, Myrsini Kaforou, Melissa Shea Hamilton, Jethro A. Herberg, Erica G. Schmitt, Agusti Rodriguez-Palmero, Aurora Pujol, Jihoon Kim, Aurélie Cobat, Laurent Abel, Shen-Ying Zhang, Jean-Laurent Casanova, Taco W. Kuijpers, Jane C. Burns, Michael Levin, Adrian C. Hayday, Vanessa Sancho-Shimizu

**Affiliations:** 1 Section of Paediatric Infectious Disease, Department of Infectious Disease, Faculty of Medicine, Imperial College London, London, UK; 2 Centre for Paediatrics and Child Health, Faculty of Medicine, Imperial College London, London, UK; 3 Section of Virology, Department of Infectious Disease, Faculty of Medicine, Imperial College London, London, UK; 4 Peter Gorer Department of Immunobiology, School of Immunology & Microbial Sciences, King’s College London, London, UK; 5 Immunosurveillance Laboratory, The Francis Crick Institute, London, UK; 6 Laboratory of Human Genetics of Infectious Diseases, Necker Branch, INSERM U1163 Necker Hospital for Sick Children, Paris, France; 7 Imagine Institute, Université Paris Cité, Paris, France; 8 Department of Pediatrics, Kawasaki Disease Research Center, University of California San Diego, La Jolla, CA, USA; 9 Rady Children’s Hospital-San Diego, San Diego, CA, USA; 10 Department of Pediatric Immunology, Rheumatology and Infectious Disease, Emma Children’s Hospital, Amsterdam University Medical Center (AmsterdamUMC), University of Amsterdam, Amsterdam, The Netherlands; 11 Department of Immunology, Institute of Biomedical Sciences, University of Sao Paulo, Sao Paulo, Brazil; 12 Department of Paediatrics and Adolescent Medicine, LKS Faculty of Medicine, The University of Hong Kong, Pokfulam, Hong Kong; 13 Department of Pediatrics, Regional University Hospital of Málaga, IBIMA Research Institute, Málaga, Spain; 14 Department of General Pediatrics and Infectious Diseases, Necker-Enfants Malades University Hospital, AP-HP, Université Paris Cité, Paris, France; 15 Hospital Universitario Marqués de Valdecilla, Santander, Spain; 16 Laboratory of Immunogenetics of Pediatric Autoimmune Diseases, INSERM UMR 1163-Institut Imagine, Paris, France; 17 Imagine Institute, Paris Descartes-Sorbonne Université Paris Cité, Paris, France; 18 Intensive Care Unit, AP-HP Pitié-Salpêtrière Hospital, Paris University, Paris, France; 19 Ghent Univer-sity Hospital, Ghent, Belgium; 20 Aix-Marseille University, APHM, Marseille, France; 21 Biocon Bristol Myers Squibb Research and Development Center, Syngene Intl. Ltd., Bengaluru, India; 22 Bristol Myers Squibb, Lawrenceville, NJ, USA; 23 Emory University Department of Pediatrics and Human Genetics, Atlanta GA, USA; 24 CHU Sainte-Justine Azrieli Research Center, Montreal, Canada; 25 Department of Microbiology, Infectious Diseases and Immunology, University of Montreal, Montreal, Canada; 26 Department of Pediatrics, University of Montreal, Montreal, Canada; 27 Division of Pediatric Infectious Diseases, Medical Faculty, Necmettin Erbakan University, Konya, Turkey; 28 Division of Pediatric Allergy and Immunology, Meram Medical Faculty, Necmettin Erbakan University, Konya, Turkey; 29 Division of Pediatric Infectious Diseases, Department of Pediatrics, Selcuk University Faculty of Medicine, Konya, Turkey; 30 Department of General Paediatrics, Hôpital Bicêtre, AP-HP, University of Paris-Saclay, Le Kremlin-Bicêtre, France; 31 Pediatric Infectious Disease and Pediatric Immunology Department, Shupyk National Healthcare University, Kyiv, Ukraine; 32 Department of Pediatric Genetics, Umraniye Education and Research Hospital, Health Sciences University, İstanbul, Turkey; 33 Division of Pediatric Rheumatology, Umraniye Training and Research Hospital, University of Health Sciences, Istanbul, Turkey; 34 Department of Pediatric Infectious Diseases, Ankara City Hospital, Ankara, Turkey; 35 Yildirim Beyazit University, Ankara City Hospital, Ankara, Turkey; 36 Division of Pediatric Critical Care Medicine, Department of Pediatrics, Faculty of Medicine, Karadeniz Technical University, Trabzon, Turkey; 37 Pediatric Infectious Diseases Unit, Bakirkoy Dr. Sadi Konuk Training and Research Hospital, University of Health Sciences, Istanbul, Turkey; 38 Department of Molecular Biology and Genetics, Bilkent University, Ankara, Turkey; 39 Service de Rhumatologie Pédiatrique, Hôpital Femme-Mère-Enfant, Groupement Hospitalier Est – Bâtiment “Pinel”, Bron, France; 40 CBC BIOTEC Biobank, GHE, Hospices Civils de Lyon, Lyon, France; 41 La Timone Children Hospital, Aix-Marseille University, APHM, Marseille, France; 42 Univeristy of Health Sciences Turkiye Bakirkoy Dr. Sadi Konuk Research and Training Hospital Pediatirc Intensive Care Department, Istanbul, Türkiye; 43 Division of Pediatric Infectious Disease, Department of Pediatrics, Faculty of Medicine, Karadeniz Technical University, Trabzon, Turkey; 44 Department of Immunology, 2nd Faculty of Medicine, Charles University in Prague and Motol University Hospital, Prague, Czech Republic; 45 Department of Paediatrics, 1st Faculty of Medicine, Charles University in Prague and Thomayer University Hospital, Prague, Czech Republic; 46 College of Medicine, Imam Abdulrahman Bin Faisal University, Dammam, Saudi Arabia; 47 Department of Pediatrics, King Fahad Hospital of the University, Al-Khobar, Saudi Arabia; 48 Department of Biochemistry, College of Medicine, Imam Abdulrahman Bin Faisal University, Dammam, Saudi Arabia; 49 Department of Pediatrics, Immunology, Infectious, and Rare Diseases at the International European University, Kyiv, Ukraine; 50 Department of Pediatric Infectious Disease, SBU Ankara Dr. Sami Ulus Maternity Child Health and Diseases Training and Research Hospital, Ankara, Turkey; 51 Department of Pediatric Infectious Diseases, Gazi University School of Medicine, Ankara, Turkey; 52 Department of Pediatrics - PICU, Faculty of Medicine in Pilsen, Charles University in Prague, Prague, Czech Republic; 53 Department of Pediatrics, 2nd Faculty of Medicine, Charles University in Prague and Motol University Hospital, Prague, Czech Republic; 54 Department of Pediatircs, Germans Trias i Pujol Research Institute, Universitat Autònoma de Barcelona, Barcelona, Spain; 55 St. Giles Laboratory of Human Genetics of Infectious Diseases, Rockefeller Branch, The Rockefeller University, New York, NY, USA; 56 Howard Hughes Medical Institute, Rockefeller University, New York, NY, USA; 57 Department of Pediatrics, Necker Hospital for Sick Children, Paris, France; 58 Paediatric Intensive Care, Evelina London Children’s Hospital, Guy’s and St Thomas’ NHS Foundation Trust, London, UK; 59 Department of Women and Children’s Health, School of Life Course Sciences, King’s College London, St Thomas’ Hospital, London, UK; 60 Paediatric Intensive Care Unit, Great Ormond Street Hospital for Children NHS Foundation Trust and NIHR Biomedical Research Centre, London, UK; 61 University College London Great Ormond St Institute of Child Health, London, UK; 62 Laboratory of Immunogenetics of Human Diseases, IdiPAZ Institute for Health Research, University Hospital “La Paz”, Madrid, Spain; 63 Centre Host Microbiome Interactions, Faculty of Dentistry, Oral & Craniofacial Sciences, King’s College London, Guy’s Tower, Guy’s Hospital, London, UK; 64 Department of Infectious Disease, Imperial College London, London, UK; 65 Division of Infection and Immunity, University College London, London, UK; 66 Helix, San Mateo, CA, USA; 67 Division of Rheumatology and Immunology, Department of Pediatrics, Washington University in St. Louis, St. Louis, MO, USA; 68 Neurometabolic Diseases Laboratory, Bellvitge Biomedical Research Institute, Barcelona, Spain; 69 Centre for Biomedical Research on Rare Diseases, Instituto de Salud Carlos III, Madrid, Spain; 70 Catalan Institution of Research and Advanced Studies, Barcelona, Spain; 71 Department of Biomedical Informatics, University of California, San Diego, CA, USA; 72 Section of Biomedical Informatics and Data Science, Yale School of Medicine, New Haven, CT, USA; 73 Department of Molecular Hematology, Sanquin Research and Landsteiner Laboratory at the AmsterdamUMC, Amsterdam Institute for Infection and Immunity, AmsterdamUMC, University of Amsterdam, Amsterdam, The Netherlands

## Abstract

Multisystem inflammatory syndrome in children (MIS-C) is a rare condition following SARS-CoV-2 infection associated with intestinal manifestations. Genetic predisposition, including inborn errors of the OAS-RNAseL pathway, has been reported. We sequenced 154 MIS-C patients and utilized a novel statistical framework of gene burden analysis, “burdenMC,” which identified an enrichment for rare predicted-deleterious variants in *BTNL8* (OR = 4.2, 95% CI: 3.5–5.3, P < 10^−6^). *BTNL8* encodes an intestinal epithelial regulator of Vγ4^+^γδ T cells implicated in regulating gut homeostasis. Enrichment was exclusive to MIS-C, being absent in patients with COVID-19 or bacterial disease. Using an available functional test for BTNL8, rare variants from a larger cohort of MIS-C patients (*n* = 835) were tested which identified eight variants in 18 patients (2.2%) with impaired engagement of Vγ4^+^γδ T cells. Most of these variants were in the B30.2 domain of BTNL8 implicated in sensing epithelial cell status. These findings were associated with altered intestinal permeability, suggesting a possible link between disrupted gut homeostasis and MIS-C-associated enteropathy triggered by SARS-CoV-2.

## Teaser

Bellos et al. identify an enrichment of rare, damaging variants in *BTNL8* in MIS-C patients. These variants result in impaired Vγ4^+^ γδ T cell engagement associated with altered gut homeostasis, which may contribute to the pathogenesis of MIS-C.

## Introduction

Severe presentation of COVID-19 in pediatric patients has remained rare throughout the SARS-CoV-2 pandemic. However, 2–6 wk after exposure, ∼1 in 10,000 such children develop a postinfectious syndrome resembling Kawasaki disease (KD), termed multisystem inflammatory syndrome in children (MIS-C) (Whittaker et al., 2020; Sancho-Shimizu et al., 2021; Dionne et al., 2022). Patients often present with similar clinical features to KD including persistent fever, rash, and conjunctivitis, although there was more frequent and profound gastrointestinal involvement (Miller et al., 2020). Epidemiological differences also differentiate KD and MIS-C, with the median age of MIS-C being 9 years compared to KD patients who are typically under 5 years of age (Sancho-Shimizu et al., 2021; Noval Rivas and Arditi, 2023), and with the incidence of KD being highest in Asia whereas MIS-C is more prevalent in Europe and the USA. Moreover, whereas the etiology for KD remains unclear, MIS-C was attributable to SARS-CoV-2 (Sancho-Shimizu et al., 2021; Noval Rivas and Arditi, 2023; Whittaker et al., 2020), with evident SARS-CoV-2 antibodies and/or a history of exposure to the virus despite there being no detectable SARS-CoV-2 infection in their upper respiratory tract upon presentation. Clinical features most commonly included fever, abdominal pain, rash, myocardial dysfunction, and elevated inflammatory biomarkers including C-reactive protein (CRP), NT-pro-BNP, ferritin, TNFα, and IL-6 (Whittaker et al., 2020; Carter et al., 2020; Gruber et al., 2020).

MIS-C is characterized by a distinct IFN-γ and NFκB-dependent signature (Sacco et al., 2022; Consiglio et al., 2020). Signatures of T cell activation and inflammation unique to MIS-C have also been detected, coupled with a specific transient polyclonal expansion of T cell receptor (TCR) Vβ21.3^+^ CD4^+^ and CD8^+^ T cells (Sacco et al., 2022; Moreews et al., 2021; Porritt et al., 2021; Jackson et al., 2023; Hoste et al., 2022). Low T cell and natural killer cell numbers have been reported, with the latter having elevated expression of cytotoxicity genes (Moreews et al., 2021; Gruber et al., 2020). Increased plasma IL-18 and surface expression of CD64 are suggestive of monocyte and neutrophil activation, respectively, in the acute phase of MIS-C (Carter et al., 2020). The mechanism underlying MIS-C remains unclear yet various possibilities have been discussed including antigen persistence, T cell exhaustion, and superantigen-mediated T cell activation (Consiglio et al., 2020; Beckmann et al., 2021; Porritt et al., 2021; Hsieh et al., 2022; Yonker et al., 2021), albeit that T cell super-activation and exhaustion were likewise implicated in COVID-19 pathology that presents very differently (Laing et al., 2020). A genetic predisposition has also been considered on the basis of epidemiological evidence, with a low incidence of MIS-C reported in East Asian (EAS) countries despite a high number of COVID-19 cases (Sancho-Shimizu et al., 2021; Carter et al., 2020; Esposito and Principi, 2021).

Monogenic inborn errors of immunity (IEI) confer increased susceptibility to infections and inflammatory diseases and have been shown in many instances to underlie COVID-19 pneumonia consequent to the ineffective production of the type I IFN response (Bousfiha et al., 2022; Zhang et al., 2020; Casanova and Anderson, 2023). A genetic predisposition has also been reported to underlie susceptibility to MIS-C in previously healthy children (Lee et al., 2020, 2023; Sancho-Shimizu et al., 2021). Rare biallelic variants impairing the 2′5′oligoadenylate synthetase-ribonuclease L pathway have been shown to lead to exacerbated inflammatory responses in 1% of MIS-C patients, owing to an inability to regulate the mitochondrial antiviral-signaling protein-mediated cytokine response in mononuclear phagocytes (Lee et al., 2023). Additionally, several children with existing IEIs with variants in *CFH*, *UNG*, and *TREX1* have reportedly developed MIS-C (Abolhassani et al., 2022). Other reported genetic risk factors for MIS-C include variants in *XIAP*, *CYBB*, and *SOCS1* which were shown to impair the negative regulation of IFN and inflammatory signaling (Chou et al., 2021; Lee et al., 2020). Additionally, enrichment of *NUMB* and *NUMBL* variants have been identified that seem to disrupt T cell function consequent to Notch1 upregulation (Benamar et al., 2023). Here, we exome-sequenced a new cohort of 154 MIS-C patients and carried out gene burden analysis to further identify genes that underlie susceptibility to MIS-C.

## Results

### Characterization of the MIS-C cohort

Pediatric patients fulfilling the WHO criteria for MIS-C were recruited from the UK, the Netherlands, and the USA between March 2020 and December 2021 (WHO, 2020). The clinical and demographic characteristics of 154 recruited MIS-C patients are summarized in Table 1. Features of the MIS-C cohort were similar to those previously described with a median age of 9 years, predominately SARS-CoV-2 IgG seropositive (81.2%) and presenting with non-specific symptoms such as fever and gastrointestinal (GI) discomfort (79.2%) (Sancho-Shimizu et al., 2021; Hoste et al., 2021; Sacco et al., 2022). There were 63.6% of patients requiring intensive care with one death. Our cohort had diverse ancestry comprising 29.2% Hispanic/American (AMR), 26.0% European (EUR), 26.6% African (AFR), 11.7% South Asian (SAS), and 3.2% EAS. These patients were whole-exome sequenced (WES), and their exomes were screened for rare non-synonymous variants that have been previously reported in IEIs (Bousfiha et al., 2022). Pathogenic variants identified and consistent with the mode of inheritance as reported by Bousfiha et al. (2022) and genotypic International Union of Immunogical Societies (IUIS) companion paper (Tangye et al., 2022) are reported in Table S2 (*CASP10*, *CD46*, *FANCC*, *PRF1*, and *TBX1*). Variants in *TBX1* have been previously reported in MIS-C but the impact remains unclear (Abolhassani et al., 2022). These variants may indeed increase the risk of developing MIS-C however further functional and clinical assessment is required to fully implicate them. Patient exomes were also screened for rare non-synonymous variants in the OAS-RNAseL pathway as biallelic variants in this pathway have been shown to lead to exacerbated inflammatory responses in MIS-C patients (Lee et al., 2023). Only monoallelic variants were identified in our cohort (Table S3).

**Table 1.** Demographic and clinical features of MIS-C cohort

	MIS-C (*n* = 154)	MIS-C with *BTNL8* variants (*n* = 9)	MIS-C with IEI variants (*n* = 6)
**Demographics**	*n* (%)	*n* (%)	*n* (%)
Sex, males	88 (57.1)	4 (44.4)	6 (100)
Age, years, median (IQR)^a^	9 (5–12)	10 (6–12)	7 (6–10)
Comorbidities	51 (33.1)	2 (22.2)	2 (33.3)
**Genetically predicted ancestry**			
AMR	45 (29.2)	2 (22.2)	1 (16.7)
AFR	41 (26.6)	3 (3.3)	3 (50)
EUR	40 (26.0)	3 (3.3)	1 (16.7)
SAS	18 (11.7)	1 (11.1)	0
EAS	5 (3.2)	0 (0)	1 (16.7)
Unknown	5 (3.2)	0 (0)	0
**Association with SARS-CoV-2**			
PCR positive	21 (13.6)	1 (11.1)	0 (0)
Serology positive	125 (81.2)	6 (66.7)	6 (100)
Serology unknown	13 (8.4)	2 (22.2)	0 (0)
**Presenting symptoms**			
Fever	154 (100)	9 (100)	6 (100)
GI	122 (79.2)	9 (100)	6 (100)
Conjunctivitis	99 (64.3)	8 (88.9)	5 (83.3)
Rash	87 (56.5)	6 (66.7)	5 (83.3)
Systemic shock	46 (29.9)	4 (44.4)	2 (33.3)
Cardiovascular	45 (29.2)	2 (22.2)	1 (16.7)
Lymphadenopathy	26 (16.9)	1 (11.1)	0 (0)
Inflamed extremities	21 (13.6)	2 (22.2)	2 (33.3)
Respiratory	16 (10.4)	0 (0)	0 (0)
Mucositis	14 (9.1)	0 (0)	0 (0)
**Severity**			
ICU	98 (63.6)	5 (55.6)	4 (66.7)
Death	1 (0.6)	0 (0)	0 (0)

a IQR, Interquartile range.

### Gene burden testing

**Figure S1.**
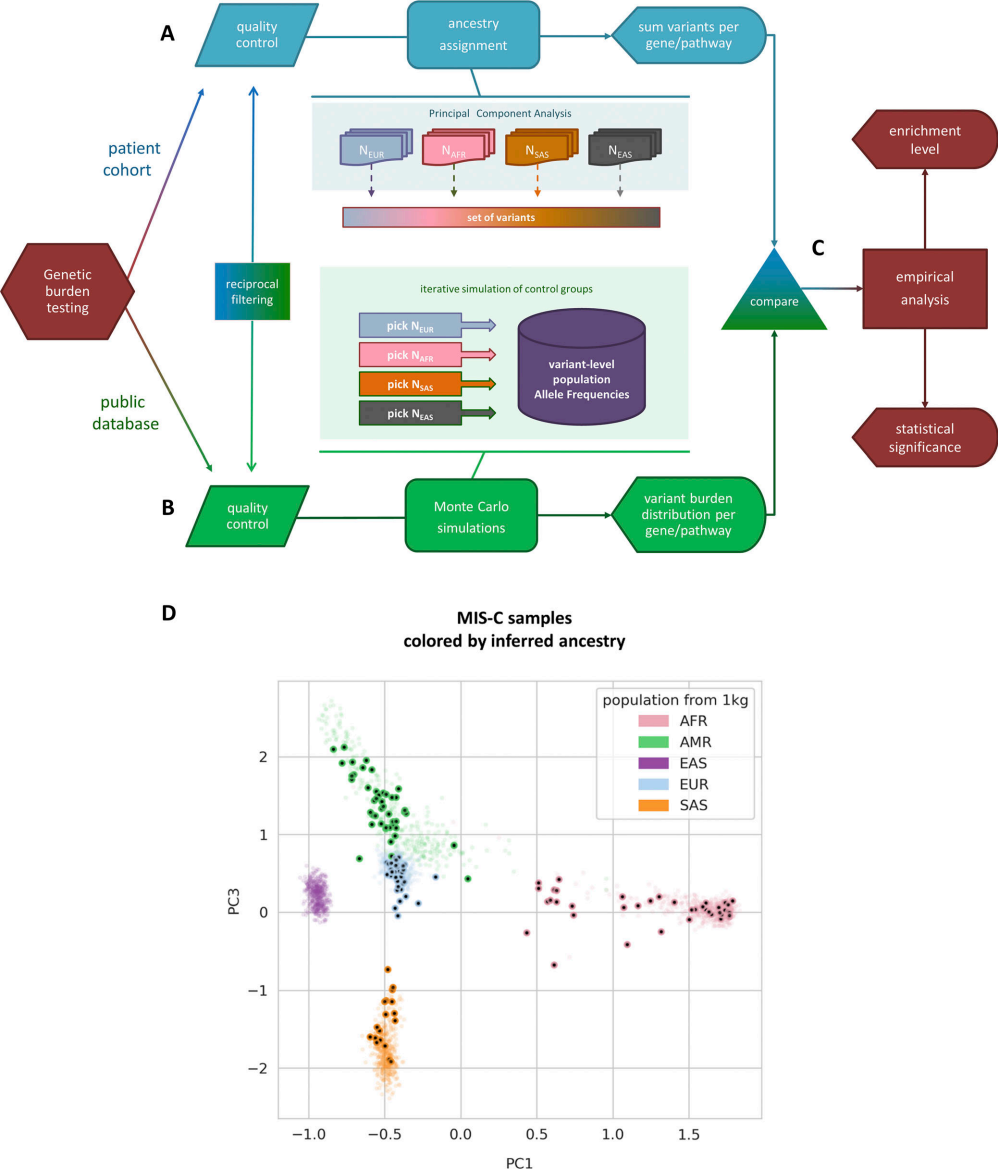
**Schematic of novel genetic burden testing method. (A)** The top part of the flowchart represents the cohort-specific analysis steps, including ancestry assignment and variant aggregation. **(B)** The bottom part of the flowchart represents the Monte Carlo simulations of control datasets using population-level AFs from public databases. Stringent quality control and variant filtering is performed both at the cohort and the database level. Variants that don’t pass the criteria in either dataset are automatically excluded from all downstream analyses. **(C)** The cohort-specific results are compared to the empirical burden distribution to estimate statistical significance. **(D)** PCA plot that depicts the inferred ancestry of all samples in the MIS-C burden analysis.

We deployed our novel statistical framework, burdenMC, to perform rare variant enrichment analysis to discover genes that may underly the susceptibility to MIS-C. Given the low incidence of MIS-C in the population (Sancho-Shimizu et al., 2021), we focused our burden analysis on rare non-synonymous variants (allele frequency <1% in each population group). Furthermore, we used the CADD score, a widely adopted meta-predictor of variant deleteriousness (Rentzsch et al., 2019), to stratify our analysis. To that end, we imposed a Phred-scaled CADD threshold of 20 to select the top 1% most deleterious variants in the dataset including predicted loss of function (pLOF) and nonsynonymous variants which we collectively considered as rare predicted-deleterious variants. As described in Table 1 and confirmed through principal component analysis (PCA) (Fig. S1), our cohort is highly diverse, with all major ancestral groups represented. For the purposes of rare deleterious burden testing, we focused on ancestral groups comprising at least 10 individuals, which excluded five EAS individuals (3% of the cohort) in additon to five individuals with ambiguous ancestry anssignment (Table 1). For the remaining groups, burdenMC testing was performed separately for each ancestry to account for differences in allelic distributions and then combined.

### Increased rare variant burden in *BTNL8*

**Figure 1.**
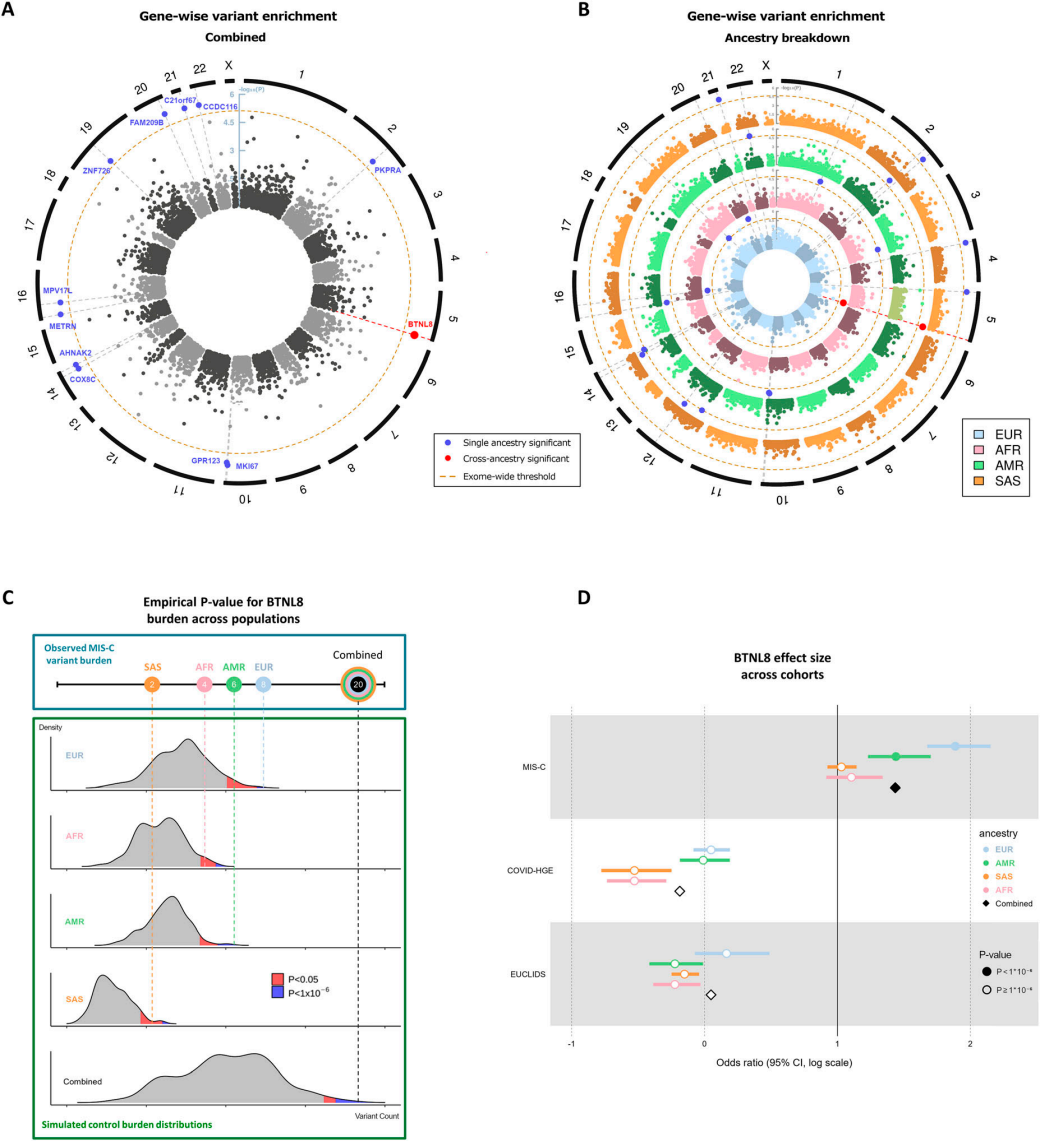
**Burden testing for rare deleterious variants in all genes across the MIS-C cohort. (A)** Circularized Manhattan plot for the combined MIS-C cohort (*n* = 144; ^*n*^EUR = 4-; *^n^*AFR = 41, ^*n*^AMR = 45, ^*n*^SAS = 18). The radial axis depicts the −log_10_(P value) of the observed burden in MIS-C, while the angular axis is arranged by genomic coordinate clock-wise. Each point in the graph represents a gene, with its radius corresponding to the empirical burden P value and its angle corresponding to the gene’s chromosomal position. The exome-wide significance threshold is denoted by the dashed inner circle. 12 genes carry a statistically significant burden of rare deleterious variants, but 11 of them are only significant in one ancestral group. **(B)** Circularized Manhattan plot for all the constituent ancestral groups in our MIS-C cohort. Each ancestry is represented in concentric plots of increasing radius to facilitate comparisons. As denoted in red, the only gene that is independently significant in multiple ancestries is *BTNL8*. **(C)** Breakdown of findings by ancestry, demonstrating the empirical P value estimation for *BTNL8*. Control burden distributions are simulated for each population based on ancestry-specific AFs and the actual sample size of the corresponding ancestral group in the MIS-C cohort. The aggregated *BTNL8* variants that are observed in MIS-C are then compared with the simulated burden distributions to determine the probability of observing a more extreme outcome. A combined P value for the entire cohort is obtained from the joint burden distribution across ancestries. *BTNL8* is statistically significant in the EUR and AMR ancestral groups (as denoted by the blue tail of the distribution) and nominally significant (AF < 0.05) in AFR and SAS (as denoted by the red tail of the distribution). As a result, *BTNL8* is also exome-wide significant in the combined analysis. **(D)** Effect size of *BTNL8* association across three cohorts. ORs for each cohort are broken down by ancestry and also include a combined effect denoted by diamonds. The ORs are depicted in log scale, with values of 0 corresponding to no difference between cases and controls. In our MIS-C cohort, rare deleterious variants in *BTNL8* appear to have a large effect, with a 4.2-fold increase in odds. This signal is largely driven by the EUR and AMR subpopulations, but all MIS-C ancestral groups have an OR > 2. In the comparator cohorts of COVID-HGE and EUCLIDS, *BTNL8* appears to have no effect with ORs around 1 across ancestries. All variants considered have an allele frequency of <1% and CADD score >20.

This analysis revealed butyrophilin-like 8 (*BTNL8*), encoding a protein expressed on healthy intestinal epithelial cells modulating intestinal Vγ4^+^γδ T cells, to be significantly enriched for rare predicted-deleterious variants at an exome-wide significance level (P < 10^−6^) (Fig. 1, A and B; and Table S4) (Di Marco Barros et al., 2016). A total allele count of 20 representing 8 variants including 1 pLOF variant was identified across 12 patients, accounting for 8.3% of our cohort. *BTNL8* was the only gene exhibiting a strong signal across multiple ancestries (Fig. 1, A–C). A further 12 genes were significantly burdened in the combined analysis, with support from only a single ancestral group (Table S4). Fig. 1, B and C demonstrates how individual ancestries contribute to the *BTNL8* enrichment. Notably, two of the largest ancestral groups in our MIS-C cohort (EUR and AMR) exhibit a standalone statistically significant burden, while the other two (AFR and SAS) are nominally significant. Based on gnomAD frequencies, burdenMC expects on average only one rare variant to be present in *BTNL8* in a cohort of the size analyzed. Of note, a variant in *BTNL8* has also been described as a risk modifier for inflammatory bowel disease (IBD) (Dart et al., 2023).

### *BTNL8* burden is not found in COVID-19 cohort

To examine whether our *BTNL8* findings were specific to MIS-C, we performed targeted burden testing in external sequencing cohorts. First, we set out to confirm that *BTNL8* is not associated with susceptibility to SARS-CoV-2 infection or severity of COVID-19. To that end, we queried the results of a large-scale rare variant burden study on COVID-19 outcomes (Butler-Laporte et al., 2022). This study meta-analyzed 21 WES and whole-genome sequencing cohorts comprising an aggregate >28,000 COVID-19 cases. In that analysis, *BTNL8* did not appear to be associated with any of the standardized COVID-19 outcomes tested, including susceptibility, severity, and hospitalization. Of note, that study utilized equivalent filters for allele frequency (AF) and deleteriousness as our MIS-C analysis and included participants from the same ancestry groups (Butler-Laporte et al., 2022) (Table S5).

### *BTNL8* burden is specific to MIS-C

To explore the role of *BTNL8* in other infectious diseases and to ascertain that the observed differential burden was not due to technical artifacts or methodological limitations, we applied burdenMC directly to two other sequencing cohorts: an in-house WES data from 502 pediatric patients with severe bacterial infections from the EUCLIDS consortium (Martinón-Torres et al., 2018), and 189 patients with COVID-19 from the COVID Human Genetic Effort (COVID-HGE) consortium (Table S1) (Casanova et al., 2020). In these datasets, burdenMC found no evidence of *BTNL8* association with bacterial disease or COVID-19. We then estimated the effect size of the *BTNL8* variant burden across these three cohorts. The results demonstrate that individuals with rare predicted-deleterious variants in *BTNL8* had a fourfold increase in the odds of presenting with MIS-C (odds ratio [OR] = 4.2, 95% confidence interval [CI]: 3.5–5.3) (Fig. 1 D). On the other hand, *BTNL8* variants did not appear to increase the odds of COVID-19 (OR = 0.8, 95% CI: 0.7–1.1) or severe bacterial infection (OR = 1.1, 95% CI: 0.8–1.4).

### Population genetics of *BTNL8* variation

**Figure S2.**
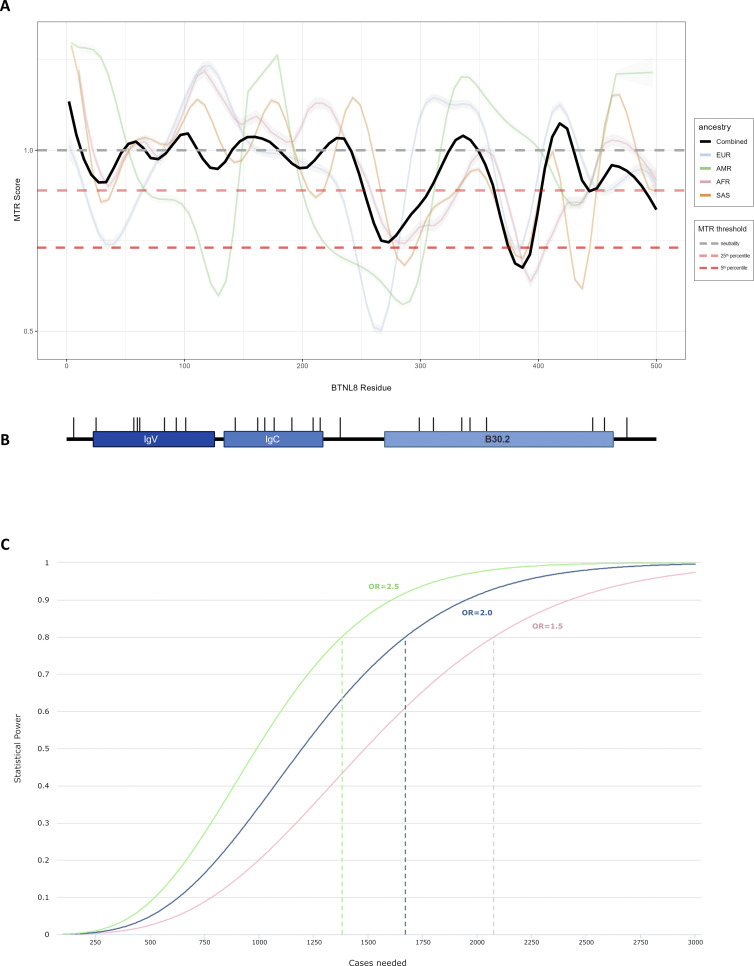
**Variation in *BTNL8*. (A)** MTR across ancestral groups calculated using gnomAD 2.1.1 data. An MTR of 1 corresponds to selective neutrality, with lower values indicating intolerance to missense variation. **(B)** Schematic of BTNL8 protein domains aligned to the coordinates of the MTR plot. **(C)** Theoretical power calculation for the BTNL8*3 CNV. Assuming a CNV AF of 26%, a large control group derived from gnomAD (100,000 individuals) and an exome-wide significance threshold of 2.5 × 10^−6^, the plot depicts the required sample size at different effect size levels. At least 1,380 MIS-C cases would be required to achieve 80% power of detecting a 2.5 OR.

As a whole, gnomAD reports *BTNL8* to be moderately intolerant to pLoF variants (LOEUF = 0.8) and missense variants (o/e = 0.83, 90% CI: 0.78–0.9) (https://gnomad.broadinstitute.org/). To estimate selective constraint at the subgenic level, we calculated the missense tolerance ratio (MTR) across *BTNL8* using data from gnomAD v2.1.1 (Silk et al., 2019). As a local measure of observed versus expected missense variation, MTR can highlight protein regions under negative selection. Although much of *BTNL8* is not predicted to deviate from neutrality (MTR = 1), there appear to be two regions under selective pressure (MTR < 0.7) across multiple ancestral groups (Fig. S2 A), both of which overlap with the intracellular B30.2 domain (Fig. S2 B). Although the precise function of the BTNL8 B30.2 domain is unresolved, such domains are common in other proteins implicated in innate immunity, wherein they mediate protein–protein interactions (Chae et al., 2006, D’Cruz et al., 2013). In this regard, the sole known function of BTNL8, which is to regulate colonic Vγ4^+^γδ T cells, totally relies on its interaction with BTNL3 (Di Marco Barros et al., 2016; Melandri et al., 2018). Additionally, the B30.2 domain of BTN3A1, a related regulator of human Vγ9^+^γδ T cells, binds low molecular phosphorylated lipids which reflect a cell’s infected and/or metabolic state (Sandstrom et al., 2014; Paysan-Lafosse et al., 2023). The genomic region encompassing *BTNL8* and *BTNL3* also contains a polymorphic copy number variant (CNV) that leads to a BTNL8*3 fusion (Aigner et al., 2013). This variant is common across ancestral groups, with an overall AF of 26% on gnomAD, and has been previously associated with a penetrating form (B3) of Crohn’s disease (Dart et al., 2023). To assess the frequency of the CNV in our cohort, we performed PCR-based CNV calling for a subset of our cohort for which there was DNA available (*n* = 115). This was undertaken using the previously reported BTNL8*3 CNV genotyping PCR (Dart et al., 2023). We did not observe a statistically significant overrepresentation of the CNV in our cohort (Table S6; AF = 22.0% in MIS-C versus 21.5% in genetic ancestry-matched controls). This may in part be due to the lack of statistical power, as relatively large sample sizes (>1,000 patients) would be required to detect differences in such common variants (Fig. S2 C).

### Protein domain burden testing

To further elucidate the effect of rare variation on functional coding elements, we also applied burdenMC to the subgenic regions corresponding to protein domains. Given the sensitivity/specificity trade-off of computational pathogenicity predictions (Sun and Yu, 2019), no CADD score filter was applied in this analysis. To that end, we selected the top 58 genes with suggestive rare variant burden (P < 0.001) and mapped 77 well annotated domains from the InterPro database (Paysan-Lafosse et al., 2023). This analysis identified domain B30.2 of *BTNL8* as the top hit (P = 1.4 × 10^−5^), with an allele count of five, representing three rare variants (AF < 1%, CADD > 0) versus none expected (Table S7). By contrast, there was no statistically significant enrichment for rare variants in the BTNL8 IgC and IgV ectodomains, for which no function has yet been established. Similarly, none of the domains mapped to the 12 other genes assessed carry a significant rare variant burden (Table S7).

### Validation of B30.2 *BTNL8* rare variant burden in independent MIS-C cohort

We set out to validate our *BTNL8* findings in an independent MIS-C cohort obtained from the COVID-HGE consortium. This cohort comprises 300 children with MIS-C that match the ancestral composition of our cohort. As a comparator control group, we acquired raw sequencing data from the ICR1000 UK exome project, comprising 1,000 control samples from the British 1958 Birth Cohort (Ruark et al., 2015). Using permutation testing, we demonstrated that the rare variant burden in the B30.2 domain of *BTNL8* was also observed in the external COVID-HGE dataset with an allele count of 8 in 300 cases (representing five variants across seven individuals; no homozygotes, no pLOF) versus 7 in 1,000 controls (P = 7 × 10^−4^), thus replicating our finding (Table S8).

### Identification of variants for functional validation

**Figure 2.**
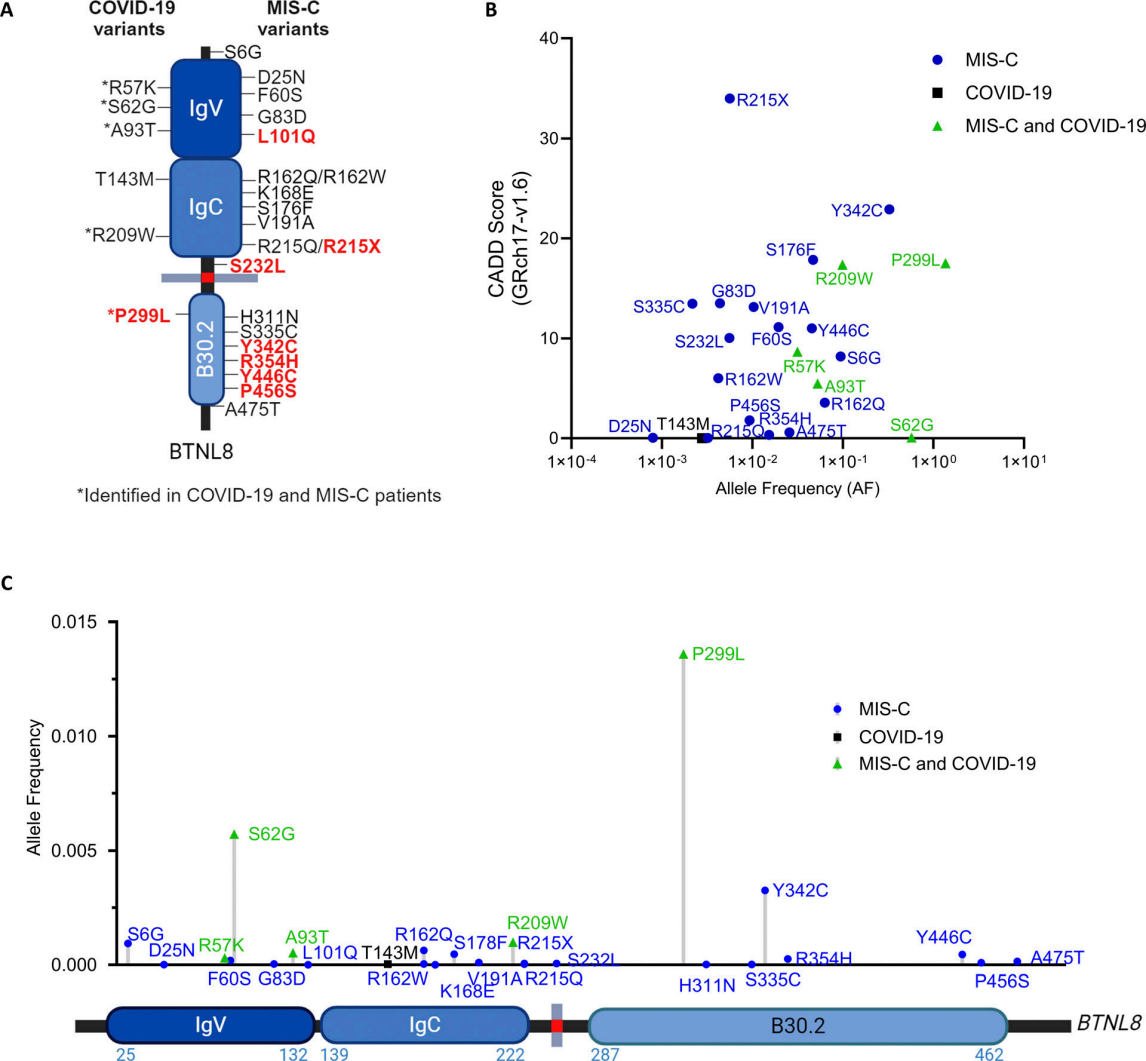
**Molecular characterization of *BTNL8* variants in MIS-C. (A)** Schematic representation of BTNL8 depicting location of identified variants. Variants with impairment indicated in red on schematic diagram. **(B)** CADD score (GRch17-v1.6) versus AF of all *BTNL8* variants (<1% AF) identified. **(C)** Lolliplot of *BTNL8* variants identified within the MIS-C and COVID-19 cohorts depicting global AF and location within the protein. p.P299L included as the variant meets the 1% cut off for several relevant ancestral groups. Images created with https://BioRender.com.

To explore the potential role(s) of *BTNL8*, we expanded the scope of our analysis to include additional MIS-C patients from external cohorts (Table 2). Following the same filtering strategy focusing on rare variants (AF < 1% & CADD > 0), we identified 21 variants in 46 patients from COVID-HGE (*n* = 690), 7 of which overlap with the variants detected in our cohort. Including the 11 variants (9 patients) in our cohort, this brings the total to 25 different variants in 55 unrelated MIS-C patients (Fig. 2, A–C and Table 2). In our MIS-C cohort, all individuals were heterozygous for the *BTNL8* variants, except for one patient with three variants (p.S6G-R162W-S176F). In the COVID-HGE MIS-C cohort, two individuals were homozygous for p.P299L and four individuals had more than one variant (p.S6G-R162Q; p.R162Q-S176F; p.P299L-A475T; p.R354H-Y446C) (Table 2). Of the 25 variants identified, seven map to the B30.2 domain. By comparison, in a control COVID-HGE-COVID19 cohort (*n* = 189), we observed only six *BTNL8* variants, five of which were also present in MIS-C (Table 2). As these have not been previously assessed functionally in the literature, we performed in silico and in vitro investigations for 26 *BTNL8* variants in total.

**Table 2.** Rare non-synonymous variants of *BTNL8* in all cohorts

dbSNP ID	Variant	Domain	AF (%)	CADD score	Functional testing	Phenotype	Genotype count^a^		
							MIS-C ICL^b^ (*n* = 146)	MIS-C COVID-HGE (*n* = 690)	COVID-19 (*n* = 189)
rs138728915	S6G	LP	0.094	8.2	Normal	MIS-C	1^c^	1^c^	
rs764166999	D25N	IgV	0.001	0.05	Normal	MIS-C		1	
rs201494649	R57K	IgV	0.031	8.68	Normal	MIS-C and COVID-19		1	2
rs751468271	F60S	IgV	0.019	11.13	Normal	MIS-C		1	
rs145199317	S62G	IgV	0.573	0.08	Normal	MIS-C and COVID-19	1	11^c^	1
rs573486977	G83D	IgV	0.004	13.52	Normal	MIS-C		2	
rs144634509	A93T	IgV	0.053	5.48	Normal	MIS-C and COVID-19	1	2	1
-	L101Q	IgV	0	20.8	Reduced	MIS-C	1		
rs749688648	T143M	IgC	0.003	0.001	Normal	COVID-19			1
rs146970792	R162Q	IgC	0.063	3.57	Normal	MIS-C	1^c^	2	
rs144634509	R162W	IgC	0.004	6.01	Normal	MIS-C	1		
-	K168E	IgC	0	21.9	Normal	MIS-C		1	
rs138408550	S176F	IgC	0.047	17.85	Normal	MIS-C	1^c^	1^c^	
rs554947046	V191A	IgC	0.01	13.15	Normal	MIS-C		6	
rs143894862	R209W	IgC	0.099	17.37	Normal	MIS-C and COVID-19	1	3	1
rs374252379	R215Q	IgC	0.003	0.04	Normal	MIS-C		1	
rs146434143	R215X	IgC	0.006	34	Loss	MIS-C		1	
rs368136851	S232L	-	0.006	10.05	Reduced	MIS-C		1	
rs151174174	P299L	B30.2	1.360^d^	17.52	Reduced	MIS-C and COVID-19	*1*	5+2(−/−)^c^	2(*−*/*−*)
rs142207026	H311N	B30.2	0.002	-	Normal	MIS-C	1		
rs372387302	S335C	B30.2	0.002	13.48	Normal	MIS-C		1	
rs188974899	Y342C	B30.2	0.326	22.9	Reduced	MIS-C		3	
rs141157357	R354H	B30.2	0.026	0.58	Reduced	MIS-C		1^c^	
rs148879045	Y446C	B30.2	0.045	11.02	Reduced	MIS-C		2^c^	
rs375876659	P456S	B30.2	0.009	1.8	Reduced	MIS-C	1		
rs375021363	A475T	-	0.015	0.35	Normal	MIS-C		1^c^	

a All monoallelic unless stated otherwise (*−*/*−*).

b ICL, Imperial College London cohort.

c Six individuals carried more than one variant: S6G + R162Q; S6G + R162Q + S176F; R162Q + S176F; P299L + A475T; R354H + Y446C.

d Included as the variant meets the 1% cut off for several relevant ancestral groups.

### Structural modeling of BTNL8/BTNL3

**Figure 3.**
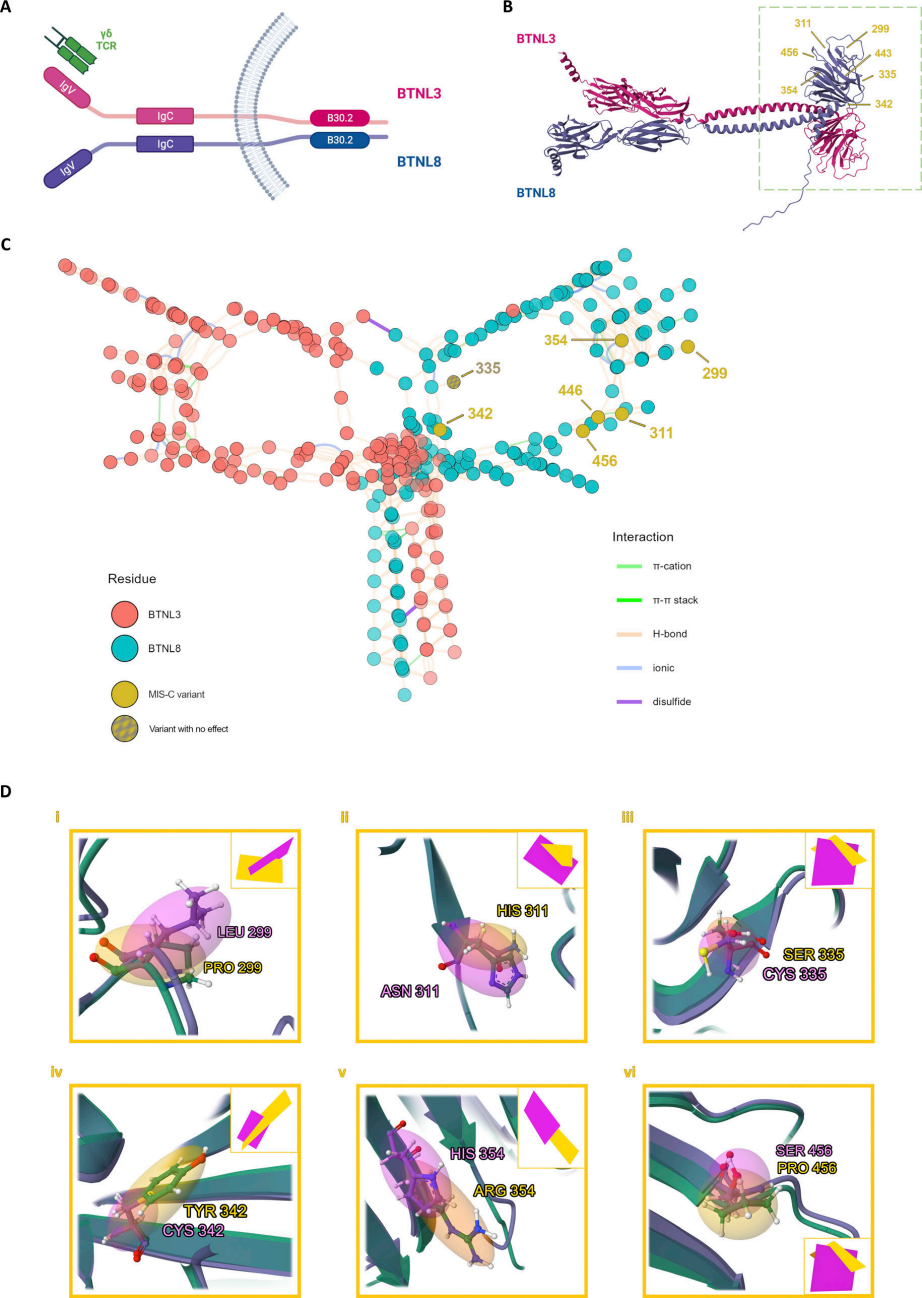
**Structural modeling of the BTNL8/BTNL3 complex. (A)** Schematic representation of the BTNL8/BTNL3 heterodimer. The intracellular B30.2 domain facilitates the stabilization of the complex. **(B)** AlphaFold-generated 3D structure prediction of the complex, visualized with Mol*. The B30.2 domain is highlighted in the green box and the location of all the variants detected in the cohort is highlighted in yellow. **(C)** RIN analysis of the B30.2 domains. Nodes in the graph correspond to residues and edges correspond to residue-residue interactions. The network is derived from the 3D structure and takes into account geometric parameters and known physico-chemical properties. The resulting graph is a 2D projection of the underlying structure, with yellow nodes indicating the positions of MIS-C variants and edge colors representing different types of interaction. **(D)** Structural modeling of B30.2 variants. AlphaFold-predicted structures for each variant (i–vi) are superimposed on the reference structure. Reference residues and corresponding 3D planes are depicted in orange, while variants are in magenta.

The structure of the BTNL8/BTNL3 complex has not been resolved, but a robust model has been proposed based on experimental data elucidating the function of the heterodimer (Melandri et al., 2018). In this model, the BTNL8–BTNL3 interface lies on the intracellular B30.2 domain which is shared by both proteins, while the TCR interaction of the complex is mediated by BTNL3’s extracellular IgV domain (Fig. 3 A). We assessed the potential effects of rare *BTNL8* variants on protein function through structural modeling of the BTNL8/BTNL3 dimer. Specifically, AlphaFold (Jumper et al., 2021) was used to generate structure predictions of the BTNL8/BTNL3 complex, and the results were visualized with Mol* (Sehnal et al., 2021) These predictions recapitulate aspects of the proposed heterodimer model while highlighting key structural features of the complex (Fig. 3). Residue interaction network (RIN) analysis of the B30.2 domain demonstrates how hydrogen bonds dominate and define the overall structure, while two disulfide bridges stabilize the complex (Fig. 3 C). Importantly, the results establish the increased connectivity of residues near the BTNL8–BTNL3 interface but also highlight additional interactions distal to the interface. Based on this analysis, five out of seven MIS-C variants in B30.2 (p.H311N, p.Y342C, p.R354H, p.Y446C, p.P456S) exhibit a high degree of residue–residue interaction.

### Protein expression of *BTNL8* variants

**Figure 4.**
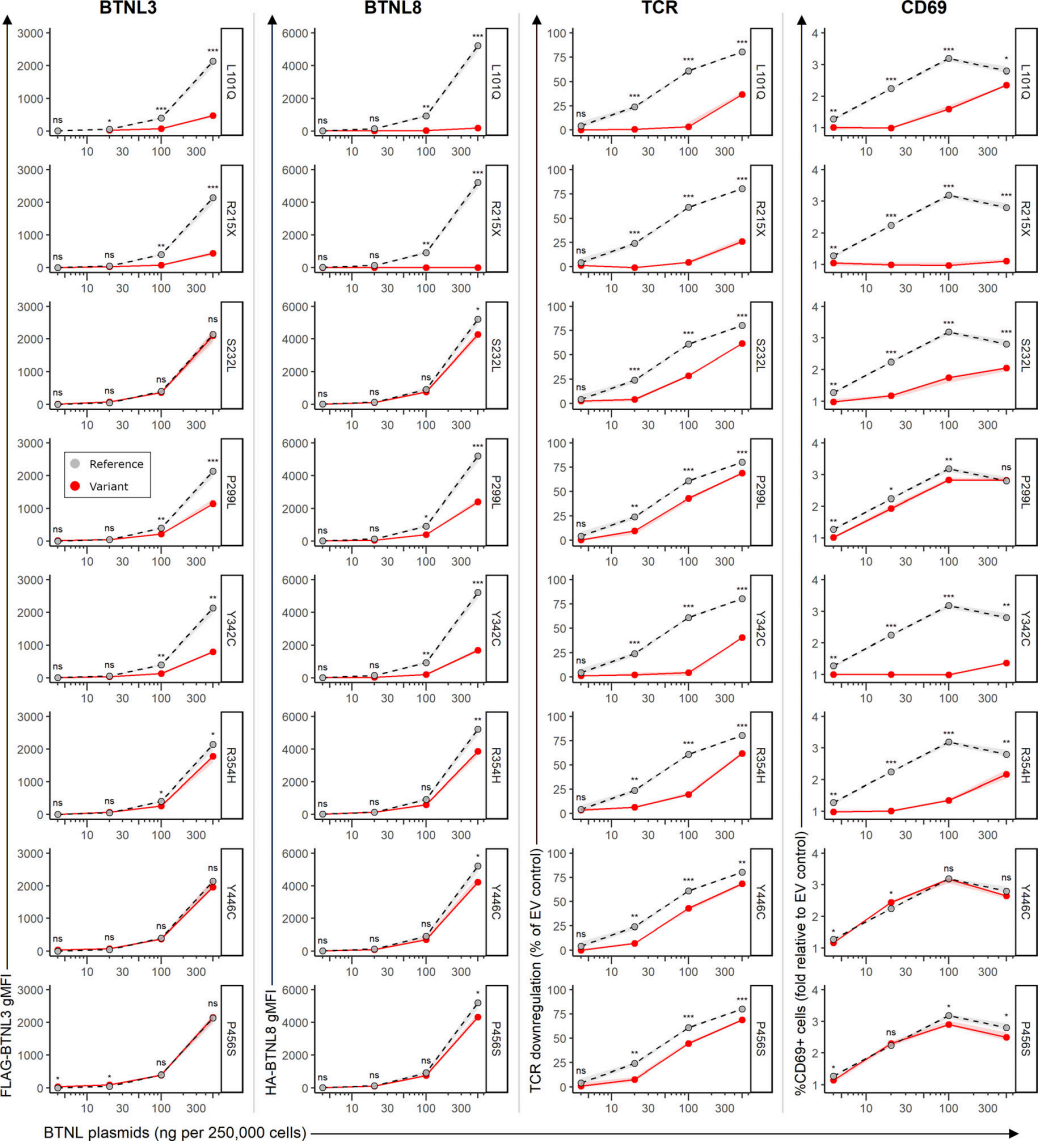
**Functional validation of BTNL8 variants.** Summary flow cytometry data of the surface expression BTNL3 (first column) and BTNL8 (second column) 48 h after transfection in 293T cells, and of TCR downregulation (third column, normalized to co-culture with cells transfected with EV control) and CD69 upregulation (fourth column, normalized to co-culture with cells transfected with EV control) by J76 cells expressing a Vg4Vg1 TCR (clone hu17) following a 5 h co-culture with transfected 293T cells. Grey symbols with dashed lines indicate WT Reference (Ref) BTNL8 sequence; red symbols with solid lines, indicate BTNL8 variants. Data points are the median of three transfections and co-cultures for each plasmid quantity. Confidence intervals (defined by the range of the replicate measurements) are represented in shaded grey and red areas. ns, not significant; *P < 0.05, **P < 0.01; ***P < 0.001 (unpaired two-tailed *t* test, comparing each BTNL8 variant to the WT Ref sequence).

**Figure S3.**
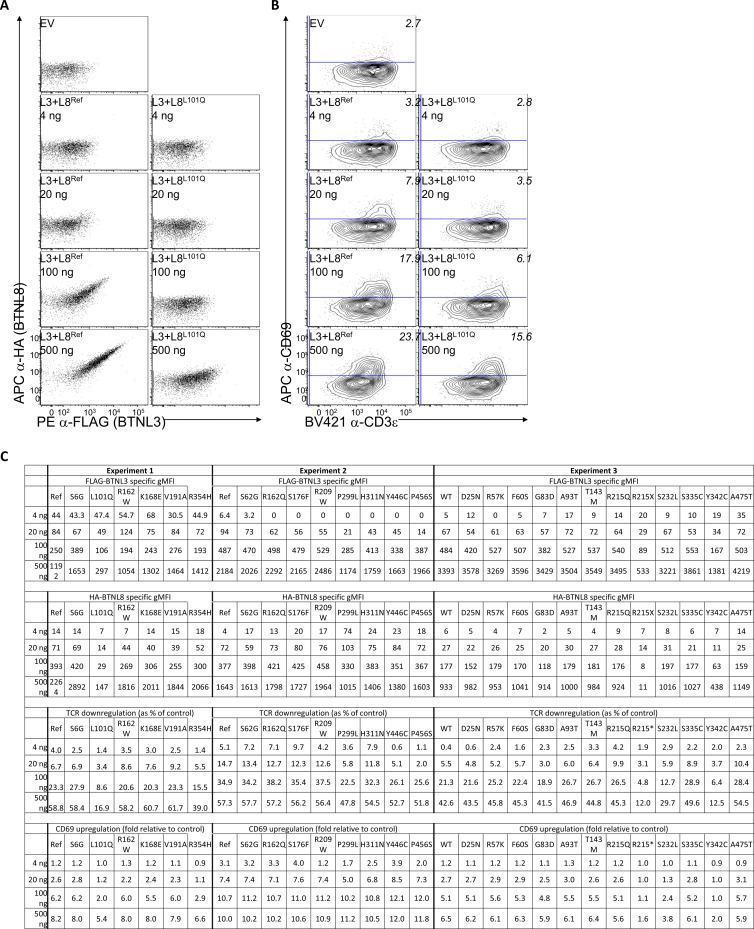
**Flow cytometry data for the functional validation of BTNL8 variants. (A and B)** Example flow cytometry plots for (A) the surface expression of BTNL3 and BTNL8 48 h posttransfection of 293T cells transfected with the indicated plasmid quantities and (B) CD3 and CD69 expression on JRT3 cells expressing a Vγ4Vδ1 TCR (clone hu17) following a 5 h co-culture with transfected 293T cells. **(C)** FLAG-BTNL3–specific gMFI, HA-BTNL8 specific gMFI, TCR downregulation (as % of control) and CD69 upregulation (fold relative to control) for all BTNL8 variants tested.

Given the co-dependence of BTNL8 and BTNL3 surface expression, changes in the cell surface expression of BTNL8 and BTNL3 were assessed following co-transfection of *BTNL8* and *BTNL3* into 293T cells which ordinarily express neither *BTNL8* nor *BTNL3*. Of the 26 variants tested, all but two variants in the IgV and IgC domains (p.L101Q and p.R215X) were expressed comparably to the WT reference allele (Fig. 4). Leucine in position 101 is highly conserved across BTN and BTNL proteins in humans and mice, and hence p.L101Q may result in improper protein folding of the variable domain, consequently impacting surface expression levels. The nonsense p.R215X mutant displayed total loss of surface expression as expected given that the premature stop codon occurs before the transmembrane domain. Only two out of seven variants in the B30.2 domain (p.H311N and p.S335C; Fig. S3) showed comparable expression levels to that of the reference WT allele, whereas variants p.P299L, p.Y342C, p.R354H, p.Y446C, and p.P456S exhibited reduced surface expression. In conclusion, of the 26 variants tested, 6 variants showed impaired cell surface expression.

### Assessing functional impact of *BTNL8* variants

We next assessed the capacity of the 26 variants to engage a Vγ4^+^ TCR, based on the established assays of TCR downregulation and CD69 upregulation, the latter reflecting the induction of TCR-dependent signaling (Fig. 4 and Fig. S3) (Melandri et al., 2018). Thus, Vγ4Vδ1-expressing Jurkat cells were co-incubated with 293T cells transduced to express the WT reference allele of *BTNL8* or variants thereof together with a WT reference allele of *BTNL3* to facilitate BTNL8 surface expression. All six variants with impaired surface expression also exhibited reduced activity with p.R215X showing complete loss of function (Fig. 4). Despite seemingly normal surface expression levels, p.S232L and p.R354H also showed impaired capacity to induce CD69 upregulation and TCR downregulation (Fig. 4). Variants p.299L, p.Y446C, and p.P456S each exhibited normal T cell activation with regards to percentage of CD69^+^ Vγ4Vδ1 cells induced but were strikingly impaired in the capacity to downregulate the TCR (Fig. 4). Of the eight variants with impaired function, most were located within the B30.2 domain (p.P299L, p.H311N, p.Y342C, p.R354H, p.Y446C, and p.P456S), highlighting its importance. In summary, we have identified 8 variants in 18 MIS-C patients with impaired BTNL8 expression and/or function among a total of 835 MIS-C patients (2.2%) This evidence suggests that in these patients, there is likely to be an impaired capacity of BTNL8/BTNL3 heterodimer to engage γδ T cells, potentially creating homeostatic imbalance in the gut, a major site of MIS-C pathology.

### *BTNL8* expression in whole blood

**Figure 5.**
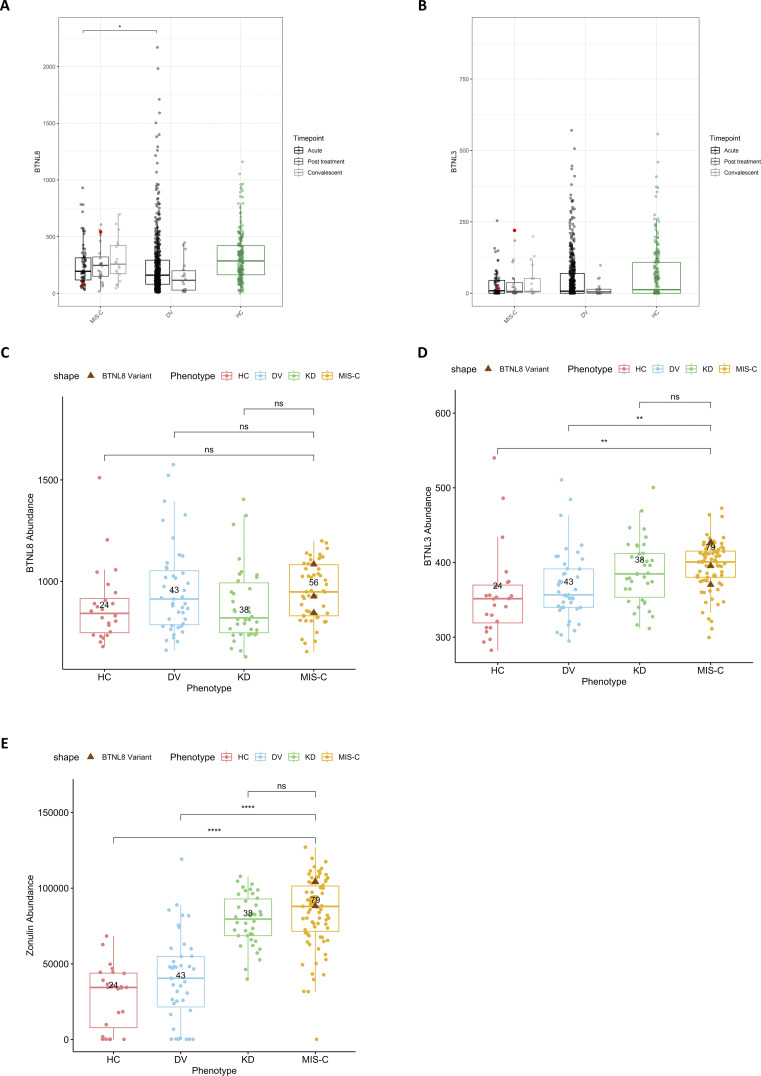
***BTNL8* and *BTNL3* expression in patient whole blood. (A and B)** *BTNL8* (A) and *BTNL3* (B) RNA expression (normalized counts) throughout disease time course. *BTNL8* expression in whole blood during acute illness (TP1), posttreatment (TP2), and convalescence (TP3) of MIS-C and DV (febrile control) phenotypes compared to healthy controls (HC). Patient carrying *BTNL8* p.P456S indicated by red dot. **(C–E)** BTNL8 (C) and BTNL3 (D) and zonulin (E) protein abundance in plasma of acute MIS-C, KD (Kawasaki Disease), and DV phenotypes compared to healthy controls. Statistical significance determined by Mann–Whitney *U* test (*P < 0.05; **P < 0.01; ****P < 0.0001).

**Figure S4.**
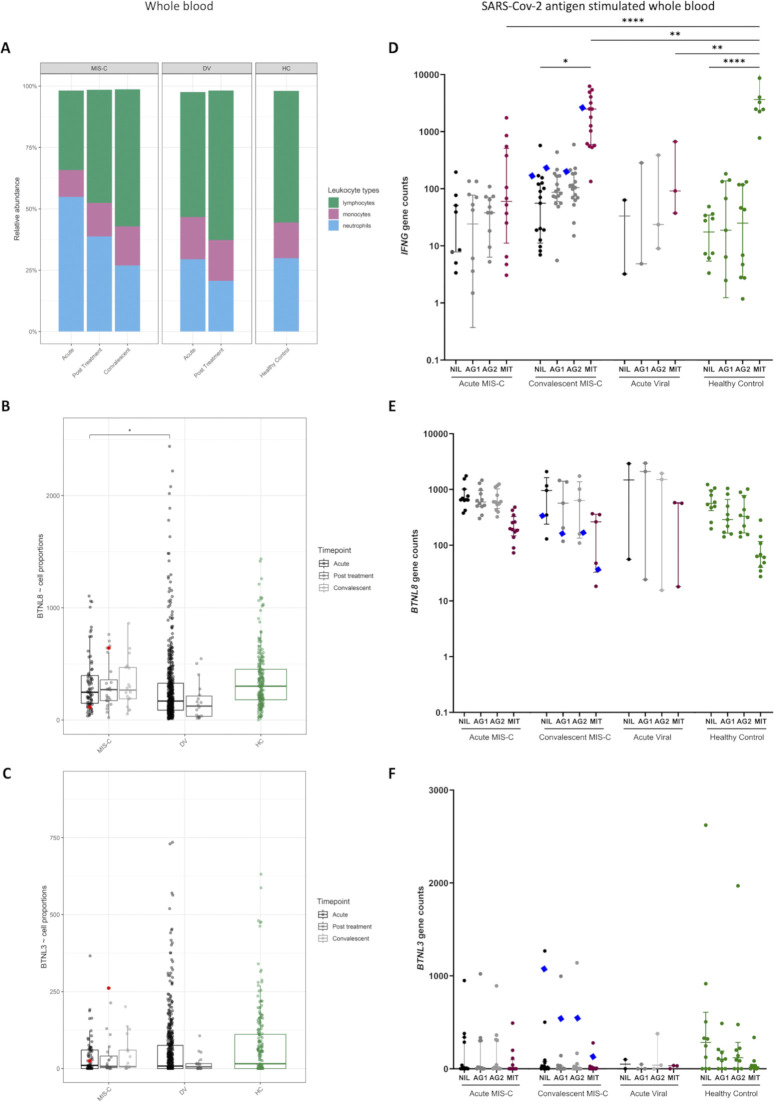
***BTNL8* expression in whole blood. (A)** Relative cellular abundances were estimated from whole blood RNA-seq data (whole blood transcriptomics cohort) using CibersortX. In the acute phase, MIS-C patients exhibit neutrophilia and lymphocytopenia, which gradually resolves. **(B)** BTNL8 expression (normalized counts) corrected for cell proportions. In the acute phase, the difference between MIS-C and viral cases survives correction. The small differences between MIS-C and healthy controls are further attenuated by the correction. **(C)** *BTNL3* expression (normalized counts) corrected for cell proportions. **(D–F)** Gene expression of *IFNG* (D), *BTNL8* (E), and *BTNL3* (F) in whole blood in response to SARS-CoV-2 antigen (QuantiFERON RNA-seq cohort). Whole blood from acute and convalescent MIS-C patients and DV phenotypes compared with healthy controls following QuantiFERON assay. Individual with *BTNL8* p.L101Q variant indicated by blue diamond. Statistical significance determined by one-way ANOVA (*P < 0.05; **P < 0.01; ****P < 0.0001).

BTNL8 is very evidently expressed exclusively by intestinal epithelial cells and also reportedly by neutrophils (https://gtexportal.org). BTNL3 is predominantly expressed in intestinal tissues and exhibits very low (arguably negligible) expression in blood. The role of BTNL8 in the gut has been extensively studied (Vantourout et al., 2018; Dart et al., 2023; Melandri et al., 2018; Willcox et al., 2019), while its role in neutrophils remains wholly unelucidated. Intestinal tissue from acute MIS-C patients was unavailable, so intestinal expression could not be assessed in our cohort. However, whole blood expression could be ascertained from existing samples, so we opted to interrogate *BTNL8* and *BTNL3* expression in an external RNA sequencing (RNA-seq) control cohort. This dataset comprises MIS-C patients (35 overlapping with our WES), pediatric healthy controls, and pediatric febrile controls (Table S1) and examines expression across three timepoints (acute illness, posttreatment, and convalescence). When compared with healthy controls, MIS-C patients did not exhibit a statistically significant difference in *BTNL8* expression (Fig. 5 A). *BTNL8* levels were significantly higher in MIS-C compared with viral febrile controls during acute illness. Furthermore, when broken down by time point within MIS-C, BTNL8 expression exhibited a trend toward decreased expression in acute illness, which recovered upon convalescence although this remains non-significant (Fig. 5 A). To determine whether this trend is driven by neutrophilia or lymphocytopenia, which are hallmarks of MIS-C (Whittaker et al., 2020; Rowley et al., 2020), we imputed relative leukocyte fractions from our RNA-seq dataset using CibersortX (Newman et al., 2019). In agreement with the literature, our MIS-C patients exhibit high neutrophil fractions in the acute phase compared with convalescence (Fig. S4 A). However, even after accounting for these differences, our *BTNL8* expression findings remain consistent (Fig. S4 B). We also examined *BTNL3* in the same RNA-seq cohort, which exhibited minimal expression across phenotypes and time points (Fig. 5 B and Fig. S4 C). In an orthogonal proteomic dataset, plasma *BTNL8* levels did not differ across disease groups (Fig. 5 C). A significant increase in BTNL3 plasma protein expression (P < 0.05) was observed in MIS-C patients (*n* = 79, including 3 of our exome-sequenced cohort; p.S6G, p.H311N; p.S6G-R162Q-S176F), although the detectible levels were so small (Fig. 5 D), that any biological significance is both unresolved and unlikely (Fig. 5, C and D).

### *BTNL8* expression upon stimulation of whole blood

Having obtained evidence that *BTNL8* expression could be detected in whole blood, we sought to evaluate changes in *BTNL8* expression that might follow exposure to SARS-CoV-2 antigens. Whole blood from an external MIS-C cohort was stimulated in SARS-CoV-2 QuantiFERON collection tubes using CD4^+^ specific SARS-CoV-2 receptor binding domain peptides (Ag1), CD4^+^/CD8^+^ specific SARS-CoV-2 peptides (Ag2), or mitogen. Expression was assessed by RNA-seq. Following QuantiFERON stimulation, MIS-C patients had elevated *IFNG* transcripts, encoding IFNγ, suggesting that patients’ cells were able to mount an immunological response to SARS-CoV-2 antigens and mitogen (Fig. S4 D). Upon convalescence, *IFNG* expression increased ∼10-fold compared with acute MIS-C, to a level that was similar to healthy controls. This could be an indicator of T cell exhaustion in the acute MIS-C samples, a known diagnostic feature of MIS-C (Shankar-Hari et al., 2023; Hoste et al., 2022). Although *BTNL8* expression was again detected, none of the stimuli induced a significant difference in expression levels although a downward trend was noted (these data include the patient with hypomorphic p.L101Q) (Fig. S4 E).

### Potential role of intestinal immunity and BTNL8 in MIS-C

**Figure S5.**
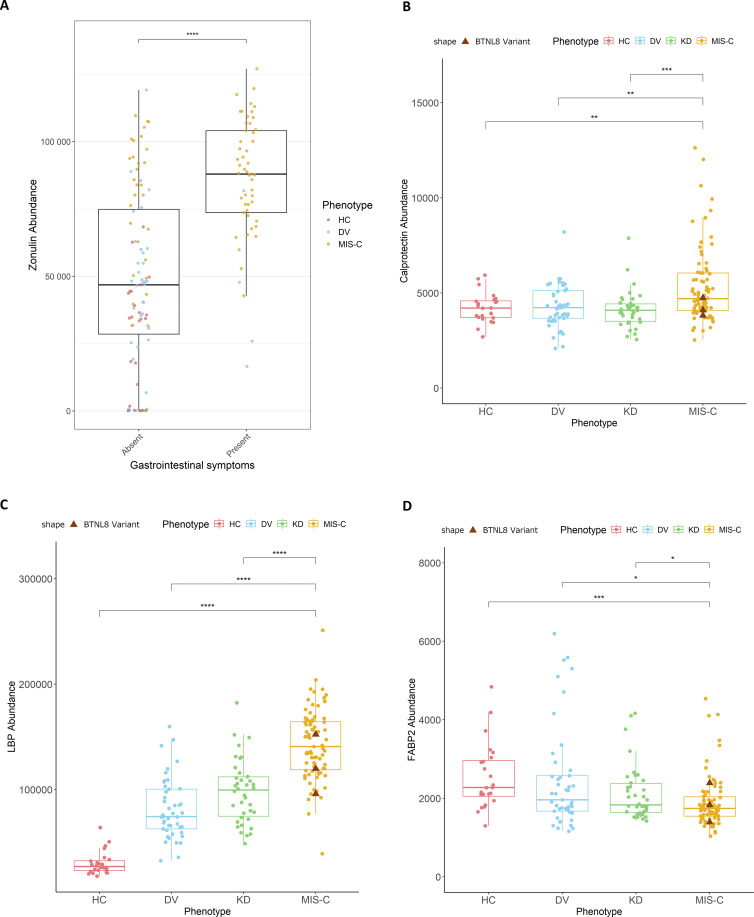
**Abundance of serum proteins used as markers for intestinal integrity. (A)** Stratified zonulin abundance comparison between individuals with reported GI symptoms and those without. Colors correspond to the underlying phenotypic group (MIS-C: patients with MIS-C; HC: healthy controls; DV: viral controls). Patients with GI symptoms exhibit higher levels of zonulin than patients without (P = 3 × 10^−10^), Mann–Whitney. **(B–D)** Calprotectin (B), lipopolysaccharide binding protein (LPB) (C), and fatty acid binding protein 2 (FABP2) (D). Abundance assessed using SomaScan in plasma of acute MIS-C, KD, and DV phenotypes compared with healthy controls. Statistical significance assessed with Mann–Whitney *U* test (four significance thresholds: *P < 0.05; **P < 0.01; ***P < 0.001; ****P < 0.0001).

**Figure 6.**
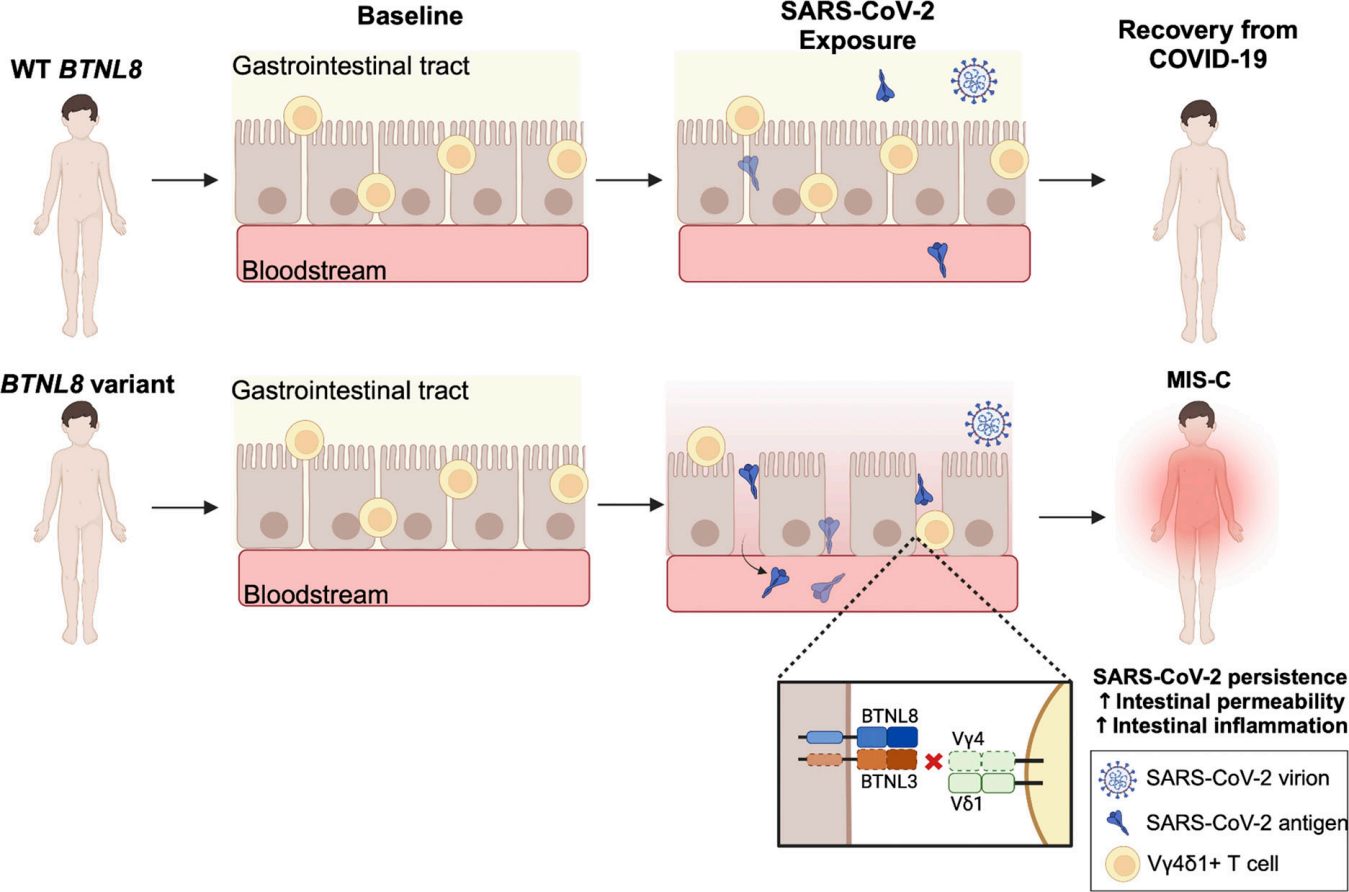
**Proposed role of *BTNL8* in MIS-C.** Schematic diagram of proposed mechanism of *BTNL8* contribution to MIS-C. Upon infection with SARS-CoV-2, *BTNL8* variants are unable to effectively engage with Vγ4^+^γδ T cells contributing to underlying intestinal inflammation subsequently leading to a hyperinflammatory state. Image created with https://BioRender.com.

The significant association of *BTNL8* variant alleles with MIS-C might offer a link to enteropathy which is a common symptom of the disease (Table 1). Interestingly, 100% of MIS-C patients with *BTNL8* variants in our cohort exhibited GI symptoms (Table 1). Among the many proposed triggers of MIS-C is the persistence of SARS-CoV-2 or SARS-CoV-2-related antigen in the gut leading to increased gut inflammation and intestinal permeability resulting possibly in antigenemia and systemic inflammation (Yonker et al., 2021). To evaluate the level of intestinal damage in MIS-C patients, in the absence of fecal samples, we assessed plasma-based intestinal permeability biomarkers including zonulin, calprotectin, LBP, and FABP2 (Kalla et al., 2016; Vreugdenhil et al., 2011; Seethaler et al., 2021; Riva et al., 2020) in MIS-C patients and controls (Fig. 5 E and Fig. S5). Increased plasma zonulin was observed in acute MIS-C and KD patients whereas increased calprotectin and LBP were found in acute MIS-C patients compared with all groups (Fig. 5 E and Fig. S5) (Yonker et al., 2021). MIS-C patients with *BTNL8* variants (*n* = 3; p.S6G, p.H311N; p.S6G-R162Q-S176F) appear to have plasma zonulin levels within the higher end of the range compared with other MIS-C patients. An increase in these biomarkers is consistent with compromised intestinal integrity. Increased FAPB2 is normally associated with intestinal permeability; however, we saw a significant decrease (Fig. S5 D), which has been reported previously in MIS-C patients with gastroenteritis (Josyabhatla et al., 2021). In addition, FABP2 is a small intestine–specific marker whereas BTNL8 is mainly associated with colonic epithelial cells potentially explaining these observations (Di Marco Barros et al., 2016; Dart et al., 2023). Moreover, previous studies have found that in adult COVID-19 patients’ expression of plasma FABP2 is especially low in patients with the highest levels of inflammation and gut permeability. Assante et al., in fact, suggested in COVID-19 patients reduced plasma FABP2 is a marker of SARS-CoV-2 infected enterocytes (Assante et al., 2022). We were unable to test pre-MIS-C or convalescent samples to assess baseline levels. However, given that baseline uninflamed gut biopsies from BTNL8*3 CNV IBD patients showed normal intestinal architecture (Dart et al., 2023), we suspected BTNL8 may not impact the baseline state but affect the resolution of inflammation once triggered. In this context, patients with variants in *BTNL8* may present with unresolved inflammation due to increased intestinal permeability and gut dysbiosis upon infection leading to systemic inflammation resulting in MIS-C (Fig. 6).

## Discussion

By applying a refined method of gene burden analysis, burdenMC, we have identified enrichment of rare genetic variants in *BTNL8* among patients with MIS-C. The burdenMC method allowed confident exome-wide enrichment analysis across a cohort of diverse ancestries. *BTNL8* variants were enriched exclusively in MIS-C when compared with genetic ancestry-matched healthy controls. We found rare predicted-deleterious variants in 8.3% of our MIS-C cohort (*n* = 144). *BTNL8* was found to be specifically associated with MIS-C and not COVID-19 or invasive bacterial diseases, contributing a fourfold increased odds of presenting with MIS-C symptoms. Notably, the *BTNL8* burden signal is driven largely by the European and Hispanic sub-cohorts, suggesting that the excess risk of *BTNL8*-related MIS-C is borne disproportionately by individuals of those ancestries. Furthermore, a protein domain burden analysis confirmed that rare variants in the B30.2 domain of *BTNL8* were enriched in our MIS-C cohort, and in an additional COVID-HGE-MIS-C cohort. We tested all 25 rare *BTNL8* variants from both MIS-C cohorts and found 8 to be functionally impaired accounting for 18 MIS-C patients affecting 2.3% of all MIS-C patients (*n* = 835). Notably, 6 of 8 functionally impaired variants were located within the B30.2 domain, which in other contexts has been shown to mediate protein-protein interactions and/or sensing of intracellular metabolites (Yuan et al., 2023; Willcox et al., 2019).

The mechanism by which these variants might promote MIS-C remains unclear. As in many inflammatory diseases, including COVID-19, MIS-C has shown dysregulation of neutrophils which reportedly express *BTNL8*. However, neither cell surface expression nor function has yet been established for *BTNL8* in neutrophils, and we had no success in identifying changes in *BTNL8* expression in whole blood from MIS-C patients effectively stimulated with SARS-CoV-2 antigens. In addition, neutrophil *BTNL8* expression has only been reported in public expression datasets (https://gtexportal.org), with no studies focused on isolated neutrophils; moreover, we did not have access to patients’ neutrophils. However, our whole blood RNA-seq data suggest that changes in neutrophil counts during acute disease did not significantly impact *BTNL8* expression (Fig. S4). Hence there is no unequivocal evidence for a role for *BTNL8* in neutrophils in our data, but this does not exclude some role for it in the development of disease; in sum, the issue warrants further investigation. In another study, BTNL8 was found to be upregulated in regulatory T cells (Tregs) and a possible marker of regulatory macrophage-induced interleukin-10-expressing-induced Tregs (Riquelme et al., 2018), which suggests another possible role for BTNL8 in the blood. Specifically, Tregs of MIS-C patients, which we were not able to assess in this study, may be an area of further investigation.

The other reported site of BTNL8 expression is the human gut epithelium where its co-expression with BTNL3 critically regulates intraepithelial Vγ4^+^ T cells that are implicated in maintaining and/or restoring the integrity of the gut barrier. Indeed, a common hypomorphic variant allele fusing *BTNL8* and *BTNL3* is associated with disease-modifying exacerbation of Crohn’s disease, consequent to dysregulation of gut Vγ4^+^ cells (Dart et al., 2023). Evidence for the importance of BTNL8 in the development of enteropathy is highlighted not only by this study suggesting a role for BTNL8 in the resolution of gut inflammation but furthermore in a recent study, where BTNL8 was used as a marker for enteropathy in immunodeficiency, polyendocrinopathy, and enteropathy X-linked syndrome (IPEX) patients (Vazquez et al., 2022). Unfortunately, intestinal tissue from acute MIS-C patients was unavailable, thus limiting our investigations. However, the prospect that the reduction in the surface expression and/or function of BTNL8 may result in hyperinflammation of the gut and increased intestinal permeability is completely consistent with increased gut permeability documented in most of our MIS-C patients, and invariably in those with variant *BTNL8* alleles. Additionally, gut barrier dysregulation may promote antigenemia and thus systemic inflammation. This is in turn is consistent with a proposed model for MIS-C in which there is a persistent reservoir of virus in the gut, demonstrated by viral RNA in stool in other studies of MIS-C (Yonker et al., 2021). The delay in onset of MIS-C following initial SARS-CoV-2 exposure as well as the persistence of virus in the intestinal milieu may reflect a breakdown in local immunological tolerance that takes time to accrue and become symptomatic. Local immunologic tolerance would be predicted to be affected by barrier integrity, consistent with which mice lacking γδ T cells show increased αβ T cell–mediated inflammation, both spontaneously and following insults (Born et al., 1999; Markle et al., 2013; Hayday and Tigelaar, 2003; Yuan et al., 2023).

Several issues remain unresolved. First, our functional assays for *BTNL8* variants are limited by our knowledge, being focused on specific aspects of Vγ4 TCR engagement that may therefore underestimate the frequency of hypomorphs among the variants. Second, although there may be many explanations, our failure to find enrichment of rare predicted-deleterious *BTNL3* variants in our cohort might also point to BTNL3-independent functions of BTNL8 that we have not assayed. Third, the striking enrichment of *BTNL8* variants in the B30.2 domain highlights the importance of better understanding its function, particularly in relation to gut epithelial cell regulation.

Finally, *BTNL8* variation has not previously been associated with an infectious disease. Given the importance of BTNL8 in regulating the severity of Crohn’s disease (Dart et al., 2023), and given animal models in which γδT cell deficiencies have been associated with disease severity rather than disease incidence, it would be of interest to further investigate the role of this pathway in pediatric IBD and other pediatric inflammatory disorders such as KD where tissue repair may be an essential limit on symptoms. Interestingly, we have observed increased zonulin levels in KD patients who have been shown to share a host immune response with MIS-C (Tsoukas and Yeung, 2022), possibly suggesting commonalities of pathogenesis (Ghosh et al., 2022; Tsoukas and Yeung, 2022). Beyond this, our data confirm that genetic susceptibility may be a contributing factor in the development of MIS-C. Understanding the genetic basis of MIS-C may not only help elucidate its pathogenesis but also provide insights in understanding and/or managing other similar hyperinflammatory syndromes.

## Materials and methods

### Human experimental guidelines approval statement

This research included patients recruited from multiple countries and was conducted with written informed consent from the residing country of the patient and in accordance with the local regulation and institutional review board approvals. These include the following: the Medical Research Guidelines (https://www.ukri.org/councils/mrc/) and the National Health Service Research Ethics Committee (https://www.hra.nhs.uk/about-us/committees-and-services/res-and-recs/), Investigating the Genetics of Infection Study REC17/LO/153; DIAMONDS: Diagnosis and Management of Febrile Illness using RNA Personalized Molecular Signature Diagnosis REC20/HRA/1714; PERFORM: Personalised Risk assessment in Febrile illness to Optimize Real-life Management across the European Union REC16/LO/1684; EUCLIDS: the genetic basis of meningococcal and other life-threatening bacterial infections of childhood REC11/LO/1982; the Medical Ethical Committee of the Amsterdam UMC NL41023.018.12; the French Ethics Committee; the French National Agency for Medicine and Health Product Safety; the Institut National de las Santé et de la Recherche Medicale in Paris, France (protocol no. C10-13); the Rockefeller University Institutional Review Board in New York, NY, USA (protocol no. JCA-0700); the University of California at San Diego Institutional Review Board (protocol no. 140220 and 170790); the University of Hong Kong/Queen Mary Hospital (protocol no. UW 20-509); and University of São Paulo (protocol no 2020/05548-8).

### Patients

MIS-C patients (*n* = 154) were enrolled via DIAMONDS (Diagnosis and Management of Febrile Illness using RNA Personalised Molecular Signature Diagnosis) (https://diamonds2020.eu) (*n* = 84); Amsterdam UMC, Netherlands (*n* = 13) and KD Research Center at University California, San Diego, La Jolla, CA, USA (*n* = 57). All ethical approvals necessary are in place for access to patient clinical data and biological samples. Clinical features of the cohort are described in Table 1, and all MIS-C patients (≤19 years old) met the World Health Organization diagnostic criteria for MIS-C (WHO, 2020) and were enrolled prior to December 2021. The diagnostic criteria as defined by the World Health Organization are as follows: Children and adolescents 0–19 years of age with fever >3 days, and two of the following: rash or bilateral non-purulent conjunctivitis or mucocutaneous inflammation signs; hypotension or shock; features of myocardial dysfunction, pericarditis, valvulitis, or coronary abnormalities; evidence of coagulopathy; and acute GI problems (diarrhea, vomiting, or abdominal pain). In addition to elevated markers of inflammation, no other obvious microbial cause of inflammation and evidence of COVID-19 or likely contact. External cohorts used for validation are summarized in Table S1. These include exome-sequenced MIS-C (*n* = 690) and SARS-CoV-2 infected children (*n* = 189) from the international COVID-HGE cohort (https://www.covidhge.com/) used for gene burden analysis; a previously published cohort of MIS-C patients (*n* = 38), healthy (*n* = 134), and febrile controls (*n* = 326) used for transcriptomic analysis recruited through DIAMONDS or prepandemic though PERFORM (Personalised Risk Assessment in Febrile Illness to Optimize Real-like Management across the European Union) (https://www.diamonds2020.eu/our-research-history/perform/) or EUCLIDS (European Union Childhood Life-Threatening Infectious disease Study (https://www.diamonds2020.eu/our-research-history/euclids/) (Jackson et al., 2023).

### WES

Genomic DNA was isolated from blood, quantified, and quality-checked before carrying out WES (Novogene Ltd.). Exome targets were captured using Agilent SureSelect Human All Exon v6, and sequenced on Illumina NovaSeq yielding 150 bp paired-end reads. The mean on-target coverage was >50×, with 95% of target bases being covered at least 10×.

### WES processing

Sequencing reads were aligned to the reference genome (hs37d5) using BWA-MEM (Li and Durbin, 2009; Li, 2013, *Preprint*). Raw-mapped reads were postprocessed to account for duplicates and systematic errors using Picard tools (https://broadinstitute.github.io/picard/). Per-sample variant calling was then performed using GATK (Van der Auwera et al., 2013; Poplin et al., 2018, *Preprint*), followed by joint genotyping across the entire cohort according to best practices. This process generated raw SNP and indel calls that were further refined and annotated using the Ensembl Variant Effect Predictor (McLaren et al., 2016). Further customized variant annotation was performed to facilitate downstream comparisons. Finally, the genotypes underwent cohort-level quality control with Peddy (Pedersen and Quinlan, 2017) to identify confounders such as cryptic relatedness and sample contamination. Genetic screening was performed to identify rare nonsynonymous variants previously reported to be involved in primary immunodeficiency (Bousfiha et al., 2022), severe COVID-19 (Zhang et al., 2020), MIS-C (Lee et al., 2023), KD, and familial hemophagocytic lymphohistiocytosis (Constantin et al., 2023) (Table S2). Medium to high-impact variants including missense, nonsense, insertion/deletion with a population AF of <0.01 were considered. Consequent to ambiguous genetically predicted ancestry assignment, nine patients were removed prior to burden testing.

### Empirical burden testing

WES is currently most informative for relatively large cohorts of ethnically homogeneous patients, who are expected to carry mutations in a small set of genes along the same biological pathway. Such cohorts are inherently underpowered to detect common variants with small effects but can be leveraged to extract knowledge from aggregated lower-frequency variants. Techniques that aggregate variants to identify genes and pathways overburdened with low-frequency mutations can help unravel the genetic etiology of uncommon disease but currently face several limitations, including, lack of matched control samples, and the requirement for ancestral homogeneity. To address these limitations, we have developed a novel computational framework, burdenMC, to extract knowledge from small, and ethnically heterogeneous patient sequencing cohorts. Our approach has been designed to be as inclusive and flexible as possible, without sacrificing statistical power.

### burdenMC

Our framework generates empirical estimates for the burden of rare variants in patient sequencing cohorts. To achieve this, we take advantage of existing large-scale population sequencing databases that capture the breadth of human genomic variation across different populations. The main contributing database is gnomAD (Gudmundsson et al., 2022), which contains whole genome and whole exome sequences of >140,000 individuals from around the globe. burdenMC is a Monte Carlo sampling method that iteratively simulates control datasets from gnomAD using only the summary statistics that are publicly available.

The process starts by determining the genetic ancestry of the individuals comprising the test cohort. To leverage the largest available training dataset, burdenMC utilizes the global ancestry inference pipeline devised by gnomAD (Karczewski et al., 2020). This entails projecting the test sequencing data onto gnomAD’s precomputed principal components and running a Random Forest classifier to assign each individual to one of five continental ancestry groups (EUR, AFR, SAS, EAS, AMR). The sizes of each ancestry group are denoted as N_EUR_, N_AFR_, N_SAS_, N_EAS_, and N_AMR_. Next, burdenMC extracts raw variant-level allele count data (AC_EUR_, AC_AFR_, AC_SAS_, AC_EAS_, and AC_AMR_) from gnomAD across the entire genome, along with the total number of gnomAD alleles in called genotypes (AN_EUR_, AN_AFR,_ AN_SAS_, AN_EAS_, and AN_AMR_). Using the hypergeometric distribution, burdenMC performs Monte Carlo resampling for each allelic variant to simulate control groups matching the test cohort. This was achieved through iterative generation of hypergeometric random variates from *Hypergeometric*(*AN*_*pop*_,*AC*_*pop*_,*N*_*pop*_), where pop∈{EUR,AFR,SAS,EAS,AMR}. To capture the extreme values of the allelic distribution, the Monte Carlo simulation was iterated 10^6^ times, thus generating 10^6^ control cohorts of size N_pop_.

For each of these control cohorts, burdenMC aggregates the number of sampled variants (at the exon, gene, pathway, or user-defined level) to obtain the empirical probability distribution of genomic burden. Finally, we calculated the variant burden in the test cohort (B_POP_) at an equivalent aggregation level and by comparing to the empirical distribution we estimate P values as *Pr*(*X*≥*B*_*POP*_), i.e., the probability of randomly observing a burden at least as large as B_POP_ (Fig. S1). To ensure comparability between the test and control sets, we performed extensive data harmonization, which includes a customized reciprocal filtering and pruning strategy.

### Variant filtering strategies

burdenMC relies on publicly available genomic datasets to simulate control samples and therefore requires rigorous quality control at the variant level. To that end, we developed a comprehensive variant filtering strategy, combining population-based metrics as well as cohort-derived statistics. A key tenet of burdenMC filtering is reciprocity, meaning if a variant is flagged in controls, it will also be filtered in cases, and vice versa. Importantly, to avoid penalizing the sensitivity of burdenMC, some of our filters are implemented as annotations (“soft filters”), with informative flags attached to the burden result to aid interpretation.

#### Population-derived filters

As it comprises the largest publicly accessible database of genomic variants, gnomAD v2.1 is used as the basis for our population filters. Variants failing gnomAD’s own Random Forest filter are removed from downstream analyses. In addition, we filter variants located in genomic regions that are not well covered in the dataset (flag: *gnomadDP0*). This is achieved by inverting gnomAD’s documented exome calling regions. Given the database’s total sample size of 141,456, we also filtered variants for which <5,000 individuals had genotype calls (flag: *ns5000*). As burdenMC depends on summarized AF, the *ns5000* filter aims to penalize sites at which rare variation may not be captured.

#### Genomic complexity filters

Certain genomic loci are challenging for both exome capture protocols and variant calling algorithms due to their low complexity. This manifests in multiple ways, most notably in the presence of repeat elements. To identify these regions, we modified the genome masking process originally devised for the Simons Genome Diversity Project (Mallick et al., 2016). Specifically, we used three criteria: the output of the symmetric DUST algorithm (score ≥ 28), which identifies tandem trinucleotide repeats (Morgulis et al., 2006); long homopolymer runs (≥7 bp); DNA satellites identified by RepeatMasker (as reported by the UCSC genome annotation database) (Smit et al., 2015). The resulting regions were merged and extended by 10 bp on either side to generate the final low-complexity region filter (flag: *lcr*).

#### Cohort-derived filters

We also implemented quality control filters that are calculated directly on the cohort being examined. We followed GATK best practices by filtering variants with potential strand bias, using the StrandOddsRatio metric (SOR > 3). We also apply a lenient filter to identify sites with excess heterozygosity (ExcessHet > 100). Similarly, we flagged variants that appear to violate Hardy-Weinberg assumptions (P value <0.001). These metrics are designed to highlight technical artifacts or cryptic consanguinity and are most informative for larger cohorts (>100 unrelated individuals).

Despite using SOR-based filtering, strand bias remained an issue particularly for genomic sites of relatively low coverage. To address this, we followed gnomAD practice by filtering variants where all individuals failed one of the following two criteria: skewed minor allele balance (<20% or >80%); low quality genotype (read depth <10 and genotype quality <20). This filter captures sites for which the only individuals that don’t exhibit strand bias are the ones with less confident genotype calls (flag: *AC0*). Finally, we excluded variants with >1% of the genotypes missing.

#### Output annotations

We also designed some annotations for the burdenMC results to highlight unusual scenarios that might require follow-up. When the burden signal is driven by only one variant, burdenMC becomes a proxy for a single-variant association test, which is not its intended purpose. The result may still be relevant to the phenotype, but further investigation is required (flag: *var1*). In another scenario, the burden signal may be driven by variants that appear to be common in the cohort of interest but are expected to be rare in the population. This may reflect a true over-representation of a causal variant, but it also captures dubious variants that have not triggered any of the previously described filters. Therefore, for variants that exhibit a pronounced allele frequency disparity between cases and controls (AF > 5% versus AF < 1%), we flag the corresponding burden result to aid interpretation (flag: *af5*).

### Linkage disequilibrium (LD) pruning

Given the breadth of genetic variation that is represented in large databases such as gnomAD, statistical methods that examine multiple genomic sites simultaneously need to account for the co-occurrence of (neighboring) variants. Especially for approaches that rely solely on summary statistics, treating each gnomAD variant as independent will result in inflated aggregate counts in the control group, thus reducing the sensitivity to detect true mutational burden. To address this challenge, we have implemented a pruning step to identify and filter variants that rely solely on summary statistics, treating each gnomAD variant as independent will result in inflated aggregate counts in the control group, thus reducing the sensitivity to detect true mutational burden. To address this challenge, we have implemented a pruning step to identify and filter variants that are in LD.To achieve this, we calculated exhaustive pairwise LD in 10-kb genomic windows using raw data of variant co-occurrence generated by gnomAD. This was performed separately per ancestral group to account for differences in LD structure. We then annotated variants exhibiting high levels of LD (r2 ≥ 0.95) for downstream pruning (flags: *ld_eur, ld_afr, ld_amr, ld_sas, ld_eas*).

Since LD patterns can be variable between populations for the same locus, pruning was not performed at the variant level, but selectively for ancestry-specific summary statistics. This process requires a memory-efficient representation of genomic variants to allow for random access of the entire gnomAD database. To that end, we employed the reversible numerical encoding formulated by VariantKey (Asuni and Wilder, 2019, *Preprint*) to create an index structure that could be queried for pruning. Using this strategy, 84,943 variants were pruned for at least one ancestral group, representing ∼1.36% of all gnomAD variants (global AF < 5%). Finally, an additional round of LD pruning is performed directly on the specific cohort being analyzed using raw sequencing data. This is designed to identify linkage patterns (r2 > 0.95) for ultra-rare and novel variants that would be absent from the databases and may thus inflate the aggregate counts in the case group, leading to type I errors.

### Effect size calculation

To quantify the effect size of aggregated variant burden, we adapted a strategy that was originally developed for the analysis of UK Biobank exome sequencing data (Backman et al., 2021; Bycroft et al., 2018).This is achieved by collapsing rare deleterious variants across the gene into a single marker. This marker is scored with 0 for individuals carrying no variants in the gene of interest, 1 for individuals carrying only heterozygous variants, and 2 for individuals carrying at least one homozygous variant. For cohorts with individual-level sequence data available, the score can be directly calculated. For comparator groups, such as gnomAD, which only provide population-level data, we utilized the Monte Carlo simulations generated by burdenMC to obtain conservative estimates of the collapsed variant score. The scores are then used as predictors in a logistic regression framework with disease status as the binary phenotype.

### Transient transfection of BTNL8 and flow cytometry

Patient variants of *BTNL8* were introduced into pCSIGPW encoding HA-BTNL8 (Vantourout et al., 2018) by site-directed mutagenesis quikchange II PCR (Agilent) and sequence confirmed. 293T human embryonic kidney cells were transfected with fivefold dilutions (4–500 ng) of HA-tagged BTNL8 and FLAG-tagged BTNL3 using PEI (3:1 PEI:DNA ratio). Total amount of transfected DNA was kept constant by supplementing BTNL plasmids with empty vector (EV) to 1 µg per 250,000 cells. 48 h posttransfection, cells were either stained with PE anti-FLAG (clone L5; Biolegend) and APC anti-HA (clone 16B12; Biolegend) to assess cell surface expression, or co-cultured for 5 h at 37°C, 5% CO_2_ with J76 cells transduced with the Vγ4Vδ1 TCR clone hu17, as previously described (Melandri et al., 2018). Co-cultured cells were then stained with PE anti-CD45 (clone HI30; Biolegend) to separate J76 and 293T cells; BV421 anti-CD3ε (clone OKT3; Biolegend) to monitor TCR downregulation; and APC anti-CD69 (clone FN50; Biolegend). To determine the surface expression of FLAG-BTNL3 and HA-BTNL8, specific geometric mean fluorescence intensities (gMFI) were calculated by subtracting the gMFI values of cells transfected with EV control and stained with anti-FLAG and anti-HA. TCR downregulation was calculated as = 100 − ([CD3ε gMFI of J76 cells co-cultured with BTNL-transfected cells]/[CD3ε gMFI of J76 cells co-cultured with EV-transfected cells]) * 100. CD69 upregulation was calculated as = (%CD69^+^ J76 cells co-cultured with BTNL-transfected cells)/(%CD69^+^ J76 cells co-cultured with EV-transfected cells).

### QuantiFERON assay and RNA-seq

An external MIS-C cohort was recruited via DIAMONDS (https://diamonds2020.eu) (QuantiFERON RNA-seq cohort; Table S1) (Shankar-Hari et al., 2023). The cohort for transcriptomic analysis included MIS-C (*n* = 21) patients with a confident viral infection phenotype (*n* = 3) and pediatric healthy controls (*n* = 10). Venous blood was collected from patients and stimulated for 16–24 h using SARS-CoV-2 QuantiFERON tubes (Qiagen) and subsequently stored in a PAXgene blood RNA tube. Total RNA was extracted using PAXgene Blood miRNA kit (Qiagen) followed by additional DNAse treatment using RNA clean & concentrator kit (Zymo Research). Bulk RNA-seq was performed using NovaSeq PE150 sequencing platform by Novogene Ltd.; generating 60 M read per sample.

### RNA-seq analysis

Prior to data analysis, genes with zero counts across all samples were removed from all datasets. PCA was performed to identify and remove outliers. Counts were normalized using DESeq2 (Love et al., 2014), and genes with zero counts across all samples post normalization were removed. Analysis of a published whole blood RNA-seq cohort (whole blood transcriptomics cohort; Table S1) was additionally performed (Jackson et al., 2023). Statistical analysis was conducted using the Mann–Whitney *U* test (P < 0.05).

### Protein structure modeling

To assess the effects of genetic variation on protein function, we developed a structural modeling framework based on AlphaFold (Jumper et al., 2021). First, structure predictions are generated using AlphaFold-multimer (Evans et al., 2022, *Preprint*) and the MMseq2-based homology search heuristic (Mirdita et al., 2022). The resulting structural models are ranked based on their template modeling scores (TM-scores) and the highest-ranking models are selected for downstream analyses and visualization. Subsequently, to identify key amino acids in the predicted structure we performed RIN analysis, using established approaches (Clementel et al., 2022). This technique employs graph theory, with amino acids represented as nodes and residue interactions represented as edges in the network. The interaction edges are defined by 3D geometry and physico-chemical properties and thus capture higher order relationships between amino acids.

### Proteomic analysis

Plasma levels of BTNL3, BTNL8, and zonulin were compared on a subset of patients analyzed on the SomaScan 7k platform (SomaLogic Inc.), a multiplexed assay that measures 7,000 known protein targets using modified aptamers (slow off-rate modified aptamers, SOMAmers), which included pediatric patients with MIS-C (*n* = 79), KD (*n* = 38), definite viral infections (DV; *n* = 43), and healthy controls (*n* = 24), recruited from the PreVAIL, DIAMONDS, and PERFORM studies (SomaScan cohort; Table S1) (Yeoh et al., 2024; Wang et al., 2023). Statistical analysis was conducted using Kruskal–Wallis and Mann–Whitney test (P < 0.05).

### Online supplemental material

Fig. S1 shows a schematic representation of our novel genetic burden testing. Fig. S2 shows the genetic variation in *BTNL8*. Fig. S3 shows example flow cytometry plots for BTNL8 and BTNL3 transfected 293T cells. Fig. S4 shows *BTNL8* gene expression in whole blood and SARS-CoV02 antigen stimulated whole blood. Fig. S5 shows abundance of serum proteins used as markers for intestinal integrity. Table S1 shows the breakdown of external cohorts described and the analyses. Table S2 shows rare non-synonymous variants in genes previously implicated in primary immunodeficiencies identified in MIS-C patients. Table S3 shows genetic variants identified in MIS-C cohort in OAS-RNAseL pathway. Table S4 shows genes significantly enriched in combined gene burden testing. Table S5 shows COVID-19 host genetics initiative meta-analysis gene burden results for BTNL8 which is adapted from supplementary material of Butler-Laporte et al. (2022). Table S6 shows CNV frequency in MIS-C cohort compared to gnomAD. Table S7 shows rare variant burden analysis at the protein domain level. Table S8 shows domain-level BTNL8 rare variant burden in COVID-HGE cohort.

## Supplementary Material

Table S1 shows the breakdown of external cohorts described and the analyses.

Table S2 shows rare non-synonymous variants in genes previously implicated in primary immunodeficiencies identified in MIS-C patients.

Table S3 shows genetic variants identified in MIS-C cohort in OAS-RNAseL pathway.

Table S4 shows genes significantly enriched in combined gene burden testing.

Table S5 shows COVID-19 Host Genetics Initiative meta-analysis gene burden results for BTNL8 (adapted from supplementary material of Butler-Laporte et al. (2022).

Table S6 shows CNV frequency in MIS-C cohort compared to gnomAD.

Table S7 shows rare variant burden analysis at the protein domain level.

Table S8 shows domain-level BTNL8 rare variant burden in COVID-HGE cohort.

## References

[bib1] Abolhassani, H., S. Delavari, N. Landegren, S. Shokri, P. Bastard, L. Du, F. Zuo, R. Hajebi, F. Abolnezhadian, S. Iranparast, . 2022. Genetic and immunologic evaluation of children with inborn errors of immunity and severe or critical COVID-19. J. Allergy Clin. Immunol. 150:1059–1073. 10.1016/j.jaci.2022.09.005 36113674 PMC9472457

[bib2] Aigner, J., S. Villatoro, R. Rabionet, J. Roquer, J. Jiménez-Conde, E. Martí, and X. Estivill. 2013. A common 56-kilobase deletion in a primate-specific segmental duplication creates a novel butyrophilin-like protein. BMC Genet. 14:61. 10.1186/1471-2156-14-61 23829304 PMC3729544

[bib3] Assante, G., A. Tourna, R. Carpani, F. Ferrari, D. Prati, F. Peyvandi, F. Blasi, A. Bandera, A. Le Guennec, S. Chokshi, . 2022. Reduced circulating FABP2 in patients with moderate to severe COVID-19 may indicate enterocyte functional change rather than cell death. Sci. Rep. 12:18792. 10.1038/s41598-022-23282-x 36335131 PMC9637119

[bib87] Asuni, N., and S. Wilder. 2019. VariantKey: A reversible numerical representation of human genetic variants. bioRxiv. 10.1101/473744 (Preprint posted February 15, 2019).

[bib4] Backman, J.D., A.H. Li, A. Marcketta, D. Sun, J. Mbatchou, M.D. Kessler, C. Benner, D. Liu, A.E. Locke, S. Balasubramanian, . 2021. Exome sequencing and analysis of 454,787 UK Biobank participants. Nature. 599:628–634. 10.1038/s41586-021-04103-z 34662886 PMC8596853

[bib5] Beckmann, N.D., P.H. Comella, E. Cheng, L. Lepow, A.G. Beckmann, S.R. Tyler, K. Mouskas, N.W. Simons, G.E. Hoffman, N.J. Francoeur, . 2021. Downregulation of exhausted cytotoxic T cells in gene expression networks of multisystem inflammatory syndrome in children. Nat. Commun. 12:4854. 10.1038/s41467-021-24981-1 34381049 PMC8357784

[bib6] Benamar, M., Q. Chen, J. Chou, A.M. Julé, R. Boudra, P. Contini, E. Crestani, P.S. Lai, M. Wang, J. Fong, . 2023. The Notch1/CD22 signaling axis disrupts Treg function in SARS-CoV-2-associated multisystem inflammatory syndrome in children. J. Clin. Invest. 133:e163235. 10.1172/JCI163235 36282598 PMC9797337

[bib7] Born, W., C. Cady, J. Jones-Carson, A. Mukasa, M. Lahn, and R. O’Brien. 1999. Immunoregulatory functions of gamma delta T cells. Adv. Immunol. 71:77–144. 10.1016/S0065-2776(08)60400-9 9917911

[bib8] Bousfiha, A., A. Moundir, S.G. Tangye, C. Picard, L. Jeddane, W. Al-Herz, C.C. Rundles, J.L. Franco, S.M. Holland, C. Klein, . 2022. The 2022 update of IUIS phenotypical classification for human inborn errors of immunity. J. Clin. Immunol. 42:1508–1520. 10.1007/s10875-022-01352-z 36198931

[bib9] Butler-Laporte, G., G. Povysil, J.A. Kosmicki, E.T. Cirulli, T. Drivas, S. Furini, C. Saad, A. Schmidt, P. Olszewski, U. Korotko, . 2022. Exome-wide association study to identify rare variants influencing COVID-19 outcomes: Results from the host genetics initiative. PLoS Genet. 18:e1010367. 10.1371/journal.pgen.1010367 36327219 PMC9632827

[bib10] Bycroft, C., C. Freeman, D. Petkova, G. Band, L.T. Elliott, K. Sharp, A. Motyer, D. Vukcevic, O. Delaneau, J. O’Connell, . 2018. The UK Biobank resource with deep phenotyping and genomic data. Nature. 562:203–209. 10.1038/s41586-018-0579-z 30305743 PMC6786975

[bib11] Carter, M.J., M. Fish, A. Jennings, K.J. Doores, P. Wellman, J. Seow, S. Acors, C. Graham, E. Timms, J. Kenny, . 2020. Peripheral immunophenotypes in children with multisystem inflammatory syndrome associated with SARS-CoV-2 infection. Nat. Med. 26:1701–1707. 10.1038/s41591-020-1054-6 32812012

[bib12] Casanova, J.L., and M.S. Anderson. 2023. Unlocking life-threatening COVID-19 through two types of inborn errors of type I IFNs. J. Clin. Invest. 133:e166283. 10.1172/JCI166283 36719370 PMC9888384

[bib13] Casanova, J.L., H.C. Su, and COVID Human Genetic Effort. 2020. A global Effort to define the human genetics of protective immunity to SARS-CoV-2 infection. Cell. 181:1194–1199. 10.1016/j.cell.2020.05.016 32405102 PMC7218368

[bib14] Chae, J.J., G. Wood, S.L. Masters, K. Richard, G. Park, B.J. Smith, and D.L. Kastner. 2006. The B30.2 domain of pyrin, the familial Mediterranean fever protein, interacts directly with caspase-1 to modulate IL-1beta production. Proc. Natl. Acad. Sci. USA. 103:9982–9987. 10.1073/pnas.0602081103 16785446 PMC1479864

[bib15] Chou, J., C.D. Platt, S. Habiballah, A.A. Nguyen, M. Elkins, S. Weeks, Z. Peters, M. Day-Lewis, T. Novak, M. Armant, . 2021. Mechanisms underlying genetic susceptibility to multisystem inflammatory syndrome in children (MIS-C). J. Allergy Clin. Immunol. 148:732–738.e1. 10.1016/j.jaci.2021.06.024 34224783 PMC8252701

[bib16] Clementel, D., A. Del Conte, A.M. Monzon, G.F. Camagni, G. Minervini, D. Piovesan, and S.C.E. Tosatto. 2022. RING 3.0: Fast generation of probabilistic residue interaction networks from structural ensembles. Nucleic Acids Res. 50:W651–W656. 10.1093/nar/gkac365 35554554 PMC9252747

[bib17] Consiglio, C.R., N. Cotugno, F. Sardh, C. Pou, D. Amodio, L. Rodriguez, Z. Tan, S. Zicari, A. Ruggiero, G.R. Pascucci, . 2020. The immunology of multisystem inflammatory syndrome in children with COVID-19. Cell. 183:968–981.e7. 10.1016/j.cell.2020.09.016 32966765 PMC7474869

[bib18] Constantin, T., T. Pék, Z. Horváth, D. Garan, and A.J. Szabó. 2023. Multisystem inflammatory syndrome in children (MIS-C): Implications for long COVID. Inflammopharmacology. 31:2221–2236. 10.1007/s10787-023-01272-3 37460909 PMC10518292

[bib19] Dart, R.J., I. Zlatareva, P. Vantourout, E. Theodoridis, A. Amar, S. Kannambath, P. East, T. Recaldin, J.C. Mansfield, C.A. Lamb, . 2023. Conserved γδ T cell selection by BTNL proteins limits progression of human inflammatory bowel disease. Science. 381:eadh0301. 10.1126/science.adh0301 37708268 PMC7615126

[bib20] Di Marco Barros, R., N.A. Roberts, R.J. Dart, P. Vantourout, A. Jandke, O. Nussbaumer, L. Deban, S. Cipolat, R. Hart, M.L. Iannitto, . 2016. Epithelia use butyrophilin-like molecules to shape organ-specific γδ T cell compartments. Cell. 167:203–218.e17. 10.1016/j.cell.2016.08.030 27641500 PMC5037318

[bib21] Dionne, A., M.B.F. Son, and A.G. Randolph. 2022. An update on multisystem inflammatory syndrome in children related to SARS-CoV-2. Pediatr. Infect. Dis. J. 41:e6–e9. 10.1097/INF.0000000000003393 34889873 PMC8658063

[bib22] D’Cruz, A.A., J.J. Babon, R.S. Norton, N.A. Nicola, and S.E. Nicholson. 2013. Structure and function of the SPRY/B30.2 domain proteins involved in innate immunity. Protein Sci. 22:1–10. 10.1002/pro.2185 PMC3575854 23139046

[bib23] Esposito, S., and N. Principi. 2021. Multisystem inflammatory syndrome in children related to SARS-CoV-2. Paediatr. Drugs. 23:119–129. 10.1007/s40272-020-00435-x 33479801 PMC7819738

[bib24] Evans, R., M. O’neill, A. Pritzel, N. Antropova, A. Senior, T. Green, A. Žídek, R. Bates, S. Blackwell, J. Yim, . 2022. Protein complex prediction with AlphaFold-Multimer. bioRxiv. 10.1101/2021.10.04.463034 (Preprint posted March 10, 2022).

[bib25] Ghosh, P., G.D. Katkar, C. Shimizu, J. Kim, S. Khandelwal, A.H. Tremoulet, J.T. Kanegaye, J. Bocchini, S. Das, J.C. Burns, . 2022. An Artificial Intelligence-guided signature reveals the shared host immune response in MIS-C and Kawasaki disease. Nat. Commun. 13:2687. 10.1038/s41467-022-30357-w 35577777 PMC9110726

[bib27] Gruber, C.N., R.S. Patel, R. Trachtman, L. Lepow, F. Amanat, F. Krammer, K.M. Wilson, K. Onel, D. Geanon, K. Tuballes, . 2020. Mapping systemic inflammation and antibody responses in multisystem inflammatory syndrome in children (MIS-C). Cell. 183:982–995.e14. 10.1016/j.cell.2020.09.034 32991843 PMC7489877

[bib28] Gudmundsson, S., M. Singer-Berk, N.A. Watts, W. Phu, J.K. Goodrich, M. Solomonson, H.L. Rehm, D.G. MacArthur, A. O’Donnell-Luria, and Genome Aggregation Database Consortium. 2022. Variant interpretation using population databases: Lessons from gnomAD. Hum. Mutat. 43:1012–1030. 10.1002/humu.24309 34859531 PMC9160216

[bib29] Hayday, A., and R. Tigelaar. 2003. Immunoregulation in the tissues by gammadelta T cells. Nat. Rev. Immunol. 3:233–242. 10.1038/nri1030 12658271

[bib30] Hoste, L., R. Van Paemel, and F. Haerynck. 2021. Multisystem inflammatory syndrome in children related to COVID-19: A systematic review. Eur. J. Pediatr. 180:2019–2034. 10.1007/s00431-021-03993-5 33599835 PMC7890544

[bib31] Hoste, L., L. Roels, L. Naesens, V. Bosteels, S. Vanhee, S. Dupont, C. Bosteels, R. Browaeys, N. Vandamme, K. Verstaen, . 2022. TIM3+ TRBV11-2 T cells and IFNγ signature in patrolling monocytes and CD16+ NK cells delineate MIS-C. J. Exp. Med. 219:e20211381. 10.1084/jem.20211381 34914824 PMC8685281

[bib32] Hsieh, L.E., J. Song, A. Grifoni, C. Shimizu, A.H. Tremoulet, K.B. Dummer, J.C. Burns, A. Sette, and A. Franco. 2022. T cells in multisystem inflammatory syndrome in children (MIS-C) have a predominant CD4+ T helper response to SARS-CoV-2 peptides and numerous virus-specific CD4− CD8− double-negative T cells. Int. J. Mol. Sci. 23:7219. 10.3390/ijms23137219 35806225 PMC9266459

[bib33] Jackson, H.R., L. Miglietta, D. Habgood-Coote, G. D’Souza, P. Shah, S. Nichols, O. Vito, O. Powell, M.S. Davidson, C. Shimizu, . 2023. Diagnosis of multisystem inflammatory syndrome in children by a whole-blood transcriptional signature. J. Pediatr. Infect. Dis. Soc. 12:322–331. 10.1093/jpids/piad035 PMC10312302 37255317

[bib34] Josyabhatla, R., A.A. Kamdar, S.A. Armbrister, R. Daniel, K. Boukas, K.G. Smith, M.R. Van Arsdall, K. Kakarala, A.R. Flores, A. Wanger, . 2021. Recognizing a MIS-chievous cause of acute viral gastroenteritis. Front Pediatr. 9:748368. 10.3389/fped.2021.748368 34778138 PMC8588082

[bib35] Jumper, J., R. Evans, A. Pritzel, T. Green, M. Figurnov, O. Ronneberger, K. Tunyasuvunakool, R. Bates, A. Žídek, A. Potapenko, . 2021. Highly accurate protein structure prediction with AlphaFold. Nature. 596:583–589. 10.1038/s41586-021-03819-2 34265844 PMC8371605

[bib36] Kalla, R., N.A. Kennedy, N.T. Ventham, R.K. Boyapati, A.T. Adams, E.R. Nimmo, M.R. Visconti, H. Drummond, G.T. Ho, R.J. Pattenden, . 2016. Serum calprotectin: A novel diagnostic and prognostic marker in inflammatory bowel diseases. Am. J. Gastroenterol. 111:1796–1805. 10.1038/ajg.2016.342 27596694

[bib37] Karczewski, K.J., L.C. Francioli, G. Tiao, B.B. Cummings, J. Alföldi, Q. Wang, R.L. Collins, K.M. Laricchia, A. Ganna, D.P. Birnbaum, . 2020. The mutational constraint spectrum quantified from variation in 141,456 humans. Nature. 581:434–443. 10.1038/s41586-020-2308-7 32461654 PMC7334197

[bib38] Laing, A.G., A. Lorenc, I. Del Molino Del Barrio, A. Das, M. Fish, L. Monin, M. Muñoz-Ruiz, D.R. McKenzie, T.S. Hayday, I. Francos-Quijorna, . 2020. A dynamic COVID-19 immune signature includes associations with poor prognosis. Nat. Med. 26:1623–1635. 10.1038/s41591-020-1038-6 32807934

[bib39] Lee, P.Y., C.D. Platt, S. Weeks, R.F. Grace, G. Maher, K. Gauthier, S. Devana, S. Vitali, A.G. Randolph, D.R. McDonald, . 2020. Immune dysregulation and multisystem inflammatory syndrome in children (MIS-C) in individuals with haploinsufficiency of SOCS1. J. Allergy Clin. Immunol. 146:1194–1200.e1. 10.1016/j.jaci.2020.07.033 32853638 PMC7445138

[bib40] Lee, D., J. Le Pen, A. Yatim, B. Dong, Y. Aquino, M. Ogishi, R. Pescarmona, E. Talouarn, D. Rinchai, P. Zhang, . 2023. Inborn errors of OAS-RNase L in SARS-CoV-2-related multisystem inflammatory syndrome in children. Science. 379:eabo3627. 10.1126/science.abo3627 36538032 PMC10451000

[bib41] Li, H. 2013. Aligning sequence reads, clone sequences and assembly contigs with BWA-MEM. arXiv. 10.48550/arXiv.1303.3997 (Preprint posted March 01, 2013).

[bib42] Li, H., and R. Durbin. 2009. Fast and accurate short read alignment with Burrows-Wheeler transform. Bioinformatics. 25:1754–1760. 10.1093/bioinformatics/btp324 19451168 PMC2705234

[bib43] Love, M.I., W. Huber, and S. Anders. 2014. Moderated estimation of fold change and dispersion for RNA-seq data with DESeq2. Genome Biol. 15:550. 10.1186/s13059-014-0550-8 25516281 PMC4302049

[bib85] Mallick, S., H. Li, M. Lipson, I. Mathieson, M. Gymrek, F. Racimo, M. Zhao, N. Chennagiri, S. Nordenfelt, A. Tandon, . 2016. The Simons Genome Diversity Project: 300 genomes from 142 diverse populations. Nature. 538:201–206. 10.1038/nature18964 27654912 PMC5161557

[bib44] Markle, J.G., S. Mortin-Toth, A.S. Wong, L. Geng, A. Hayday, and J.S. Danska. 2013. γδ T cells are essential effectors of type 1 diabetes in the nonobese diabetic mouse model. J. Immunol. 190:5392–5401. 10.4049/jimmunol.1203502 23626013 PMC3836168

[bib45] Martinón-Torres, F., A. Salas, I. Rivero-Calle, M. Cebey-López, J. Pardo-Seco, J.A. Herberg, N.P. Boeddha, D.S. Klobassa, F. Secka, S. Paulus, . 2018. Life-threatening infections in children in Europe (the EUCLIDS project): A prospective cohort study. Lancet Child Adolesc. Health. 2:404–414. 10.1016/S2352-4642(18)30113-5 30169282

[bib46] McLaren, W., L. Gil, S.E. Hunt, H.S. Riat, G.R. Ritchie, A. Thormann, P. Flicek, and F. Cunningham. 2016. The Ensembl variant effect predictor. Genome Biol. 17:122. 10.1186/s13059-016-0974-4 27268795 PMC4893825

[bib47] Melandri, D., I. Zlatareva, R.A.G. Chaleil, R.J. Dart, A. Chancellor, O. Nussbaumer, O. Polyakova, N.A. Roberts, D. Wesch, D. Kabelitz, . 2018. The γδTCR combines innate immunity with adaptive immunity by utilizing spatially distinct regions for agonist selection and antigen responsiveness. Nat. Immunol. 19:1352–1365. 10.1038/s41590-018-0253-5 30420626 PMC6874498

[bib48] Miller, J., A. Cantor, P. Zachariah, D. Ahn, M. Martinez, and K.G. Margolis. 2020. Gastrointestinal symptoms as a major presentation component of a novel multisystem inflammatory syndrome in children that is related to coronavirus disease 2019: A single center experience of 44 cases. Gastroenterology. 159:1571–1574.e2. 10.1053/j.gastro.2020.05.079 32505742 PMC7270806

[bib49] Mirdita, M., K. Schütze, Y. Moriwaki, L. Heo, S. Ovchinnikov, and M. Steinegger. 2022. ColabFold: Making protein folding accessible to all. Nat. Methods. 19:679–682. 10.1038/s41592-022-01488-1 35637307 PMC9184281

[bib50] Moreews, M., K. Le Gouge, S. Khaldi-Plassart, R. Pescarmona, A.L. Mathieu, C. Malcus, S. Djebali, A. Bellomo, O. Dauwalder, M. Perret, . 2021. Polyclonal expansion of TCR Vbeta 21.3(+) CD4(+) and CD8(+) T cells is a hallmark of multisystem inflammatory syndrome in children. Sci. Immunol. 6:eabh1516. 10.1126/sciimmunol.abh1516 34035116 PMC8815705

[bib86] Morgulis, A., E.M. Gertz, A.A. Schäffer, and R. Agarwala. 2006. A fast and symmetric DUST implementation to mask low-complexity DNA sequences. J. Comput. Biol. 13:1028–1040. 10.1089/cmb.2006.13.1028 16796549

[bib51] Newman, A.M., C.B. Steen, C.L. Liu, A.J. Gentles, A.A. Chaudhuri, F. Scherer, M.S. Khodadoust, M.S. Esfahani, B.A. Luca, D. Steiner, . 2019. Determining cell type abundance and expression from bulk tissues with digital cytometry. Nat. Biotechnol. 37:773–782. 10.1038/s41587-019-0114-2 31061481 PMC6610714

[bib52] Noval Rivas, M., and M. Arditi. 2023. Kawasaki disease and multisystem inflammatory syndrome in children: Common inflammatory pathways of two distinct diseases. Rheum. Dis. Clin. North Am. 49:647–659. 10.1016/j.rdc.2023.03.002 37331738 PMC10020039

[bib53] Paysan-Lafosse, T., M. Blum, S. Chuguransky, T. Grego, B.L. Pinto, G.A. Salazar, M.L. Bileschi, P. Bork, A. Bridge, L. Colwell, . 2023. InterPro in 2022. Nucleic Acids Res. 51:D418–D427. 10.1093/nar/gkac993 36350672 PMC9825450

[bib54] Pedersen, B.S., and A.R. Quinlan. 2017. Who’s who? Detecting and resolving sample anomalies in human DNA sequencing studies with Peddy. Am. J. Hum. Genet. 100:406–413. 10.1016/j.ajhg.2017.01.017 28190455 PMC5339084

[bib56] Poplin, R., V. Ruano-Rubio, M.A. Depristo, T.J. Fennell, M.O. Carneiro, G.V.D. Auwera, D.E. Kling, L.D. Gauthier, A. Levy-Moonshine, D. Roazen, . 2018. Scaling accurate genetic variant discovery to tens of thousands of samples. bioRxiv. 10.1101/201178 (Preprint posted July 24, 2018).

[bib57] Porritt, R.A., L. Paschold, M.N. Rivas, M.H. Cheng, L.M. Yonker, H. Chandnani, M. Lopez, D. Simnica, C. Schultheiß, C. Santiskulvong, . 2021. HLA class I-associated expansion of TRBV11-2 T cells in multisystem inflammatory syndrome in children. J. Clin. Invest. 131:e146614. 10.1172/JCI146614 33705359 PMC8121516

[bib58] Rentzsch, P., D. Witten, G.M. Cooper, J. Shendure, and M. Kircher. 2019. CADD: Predicting the deleteriousness of variants throughout the human genome. Nucleic Acids Res. 47:D886–D894. 10.1093/nar/gky1016 30371827 PMC6323892

[bib59] Riquelme, P., J. Haarer, A. Kammler, L. Walter, S. Tomiuk, N. Ahrens, A.K. Wege, I. Goecze, D. Zecher, B. Banas, . 2018. TIGIT^+^ iTregs elicited by human regulatory macrophages control T cell immunity. Nat. Commun. 9:2858. 10.1038/s41467-018-05167-8 30030423 PMC6054648

[bib60] Riva, A., E.H. Gray, S. Azarian, A. Zamalloa, M.J.W. McPhail, R.P. Vincent, R. Williams, S. Chokshi, V.C. Patel, and L.A. Edwards. 2020. Faecal cytokine profiling as a marker of intestinal inflammation in acutely decompensated cirrhosis. JHEP Rep. Innov. Hepatol. 2:100151. 10.1016/j.jhepr.2020.100151 PMC7391986 32838247

[bib61] Rowley, A.H., S.T. Shulman, and M. Arditi. 2020. Immune pathogenesis of COVID-19-related multisystem inflammatory syndrome in children. J. Clin. Invest. 130:5619–5621. 10.1172/JCI143840 32870815 PMC7598032

[bib62] Ruark, E., M. Münz, A. Renwick, M. Clarke, E. Ramsay, S. Hanks, S. Mahamdallie, A. Elliott, S. Seal, A. Strydom, . 2015. The ICR1000 UK exome series: A resource of gene variation in an outbred population. F1000 Res. 4:883. 10.12688/f1000research.7049.1 PMC4706061 26834991

[bib63] Sacco, K., R. Castagnoli, S. Vakkilainen, C. Liu, O.M. Delmonte, C. Oguz, I.M. Kaplan, S. Alehashemi, P.D. Burbelo, F. Bhuyan, . 2022. Immunopathological signatures in multisystem inflammatory syndrome in children and pediatric COVID-19. Nat. Med. 28:1050–1062. 10.1038/s41591-022-01724-3 35177862 PMC9119950

[bib64] Sancho-Shimizu, V., P. Brodin, A. Cobat, C.M. Biggs, J. Toubiana, C.L. Lucas, S.E. Henrickson, A. Belot, S.G. Tangye, J.D. Milner, . 2021. SARS-CoV-2-related MIS-C: A key to the viral and genetic causes of Kawasaki disease? J. Exp. Med. 218:e20210446. 10.1084/jem.20210446 33904890 PMC8080850

[bib65] Sandstrom, A., C.M. Peigné, A. Léger, J.E. Crooks, F. Konczak, M.C. Gesnel, R. Breathnach, M. Bonneville, E. Scotet, and E.J. Adams. 2014. The intracellular B30.2 domain of butyrophilin 3A1 binds phosphoantigens to mediate activation of human Vγ9Vδ2 T cells. Immunity. 40:490–500. 10.1016/j.immuni.2014.03.003 24703779 PMC4028361

[bib66] Seethaler, B., M. Basrai, A.M. Neyrinck, J.A. Nazare, J. Walter, N.M. Delzenne, and S.C. Bischoff. 2021. Biomarkers for assessment of intestinal permeability in clinical practice. Am. J. Physiol. Gastrointest. Liver Physiol. 321:G11–G17. 10.1152/ajpgi.00113.2021 34009040

[bib67] Sehnal, D., S. Bittrich, M. Deshpande, R. Svobodová, K. Berka, V. Bazgier, S. Velankar, S.K. Burley, J. Koča, and A.S. Rose. 2021. Mol* viewer: Modern web app for 3D visualization and analysis of large biomolecular structures. Nucleic Acids Res. 49:W431–W437. 10.1093/nar/gkab314 33956157 PMC8262734

[bib68] Shankar-Hari, M., H. Patel, M. Carter, H. Jackson, O. Powell, M. Fish, M.T. Barberio, F. Spada, N. Petrov, P. Wellman, . 2023. Immunology of severe febrile illness in children in the COVID-19 era. Res. Square. 10.21203/rs.3.rs-3385634/v1

[bib69] Silk, M., S. Petrovski, and D.B. Ascher. 2019. MTR-viewer: Identifying regions within genes under purifying selection. Nucleic Acids Res. 47:W121–W126. 10.1093/nar/gkz457 31170280 PMC6602522

[bib130] Smit, A., R. Hubley, and P. Green. 2015. RepeatMasker Open-4.0. http://www.repeatmasker.org.

[bib70] Sun, H., and G. Yu. 2019. New insights into the pathogenicity of non-synonymous variants through multi-level analysis. Sci. Rep. 9:1667. 10.1038/s41598-018-38189-9 30733553 PMC6367327

[bib71] Tangye, S.G., W. Al-Herz, A. Bousfiha, C. Cunningham-Rundles, J.L. Franco, S.M. Holland, C. Klein, T. Morio, E. Oksenhendler, C. Picard, . 2022. Human inborn errors of immunity: 2022 update on the classification from the international union of immunological societies expert committee. J. Clin. Immunol. 42:1473–1507. 10.1007/s10875-022-01289-3 35748970 PMC9244088

[bib72] Tsoukas, P., and R.S.M. Yeung. 2022. Kawasaki disease and MIS-C share a host immune response. Nat. Rev. Rheumatol. 18:555–556. 10.1038/s41584-022-00820-5 36008614 PMC9406247

[bib73] Van Der Auwera, G.A., Carneiro, M.O., Hartl, C., Poplin, R., Del Angel, G., Levy-Moonshine, A., Jordan, T., Shakir, K., Roazen, D., Thibault, J., . 2013. From FastQ data to high confidence variant calls: The genome analysis toolkit best practices pipeline. Curr. Protoc. Bioinformatics. 43, 11.10.1–11.10.33. 10.1002/0471250953.bi1110s43 PMC4243306 25431634

[bib74] Vantourout, P., A. Laing, M.J. Woodward, I. Zlatareva, L. Apolonia, A.W. Jones, A.P. Snijders, M.H. Malim, and A.C. Hayday. 2018. Heteromeric interactions regulate butyrophilin (BTN) and BTN-like molecules governing γδ T cell biology. Proc. Natl. Acad. Sci. USA. 115:1039–1044. 10.1073/pnas.1701237115 29339503 PMC5798315

[bib75] Vazquez, S.E., S.A. Mann, A. Bodansky, A.F. Kung, Z. Quandt, E.M.N. Ferré, N. Landegren, D. Eriksson, P. Bastard, S.Y. Zhang, . 2022. Autoantibody discovery across monogenic, acquired, and COVID-19-associated autoimmunity with scalable PhIP-seq. Elife. 11:e78550. 10.7554/eLife.78550 36300623 PMC9711525

[bib76] Vreugdenhil, A.C., V.M. Wolters, M.P. Adriaanse, A.M. Van den Neucker, A.A. van Bijnen, R. Houwen, and W.A. Buurman. 2011. Additional value of serum I-FABP levels for evaluating celiac disease activity in children. Scand. J. Gastroenterol. 46:1435–1441. 10.3109/00365521.2011.627447 22029621

[bib77] Wang, H., C. Shimizu, E. Bainto, S. Hamilton, H.R. Jackson, D. Estrada-Rivadeneyra, M. Kaforou, M. Levin, J.M. Pancheri, K.B. Dummer, . 2023. Subgroups of children with Kawasaki disease: A data-driven cluster analysis. Lancet Child Adolesc. Health. 7:697–707. 10.1016/S2352-4642(23)00166-9 37598693 PMC10756500

[bib78] Whittaker, E., A. Bamford, J. Kenny, M. Kaforou, C.E. Jones, P. Shah, P. Ramnarayan, A. Fraisse, O. Miller, P. Davies, . 2020. Clinical characteristics of 58 children with a pediatric inflammatory multisystem syndrome temporally associated with SARS-CoV-2. JAMA. 324:259–269. 10.1001/jama.2020.10369 32511692 PMC7281356

[bib79] WHO 2020. Multisystem inflammatory syndrome in children and adolescents temporally related to COVID-19. https://www.who.int/news-room/commentaries/detail/multisystem-inflammatory-syndrome-in-children-and-adolescents-with-covid-19 (accessed May 19, 2023).

[bib80] Willcox, C.R., P. Vantourout, M. Salim, I. Zlatareva, D. Melandri, L. Zanardo, R. George, S. Kjaer, M. Jeeves, F. Mohammed, . 2019. Butyrophilin-like 3 directly binds a human Vγ4^+^ T cell receptor using a modality distinct from clonally-restricted antigen. Immunity. 51:813–825.e4. 10.1016/j.immuni.2019.09.006 31628053 PMC6868513

[bib81] Yeoh, S., D. Estrada-Rivadeneyra, H. Jackson, I. Keren, R. Galassini, S. Cooray, P. Shah, P. Agyeman, R. Basmaci, E. Carrol, . 2024. Plasma protein biomarkers distinguish multisystem inflammatory syndrome in children from other pediatric infectious and inflammatory diseases. Pediatr. Infect. Dis. J. 43:444–453. 10.1097/INF.0000000000004267 38359342 PMC11003410

[bib82] Yonker, L.M., T. Gilboa, A.F. Ogata, Y. Senussi, R. Lazarovits, B.P. Boribong, Y.C. Bartsch, M. Loiselle, M.N. Rivas, R.A. Porritt, . 2021. Multisystem inflammatory syndrome in children is driven by zonulin-dependent loss of gut mucosal barrier. J. Clin. Invest. 131:e149633. 10.1172/JCI149633 34032635 PMC8279585

[bib83] Yuan, L., X. Ma, Y. Yang, Y. Qu, X. Li, X. Zhu, W. Ma, J. Duan, J. Xue, H. Yang, . 2023. Phosphoantigens glue butyrophilin 3A1 and 2A1 to activate Vγ9Vδ2 T cells. Nature. 621:840–848. 10.1038/s41586-023-06525-3 37674084 PMC10533412

[bib84] Zhang, Q., P. Bastard, Z. Liu, J. Le Pen, M. Moncada-Velez, J. Chen, M. Ogishi, I.K.D. Sabli, S. Hodeib, C. Korol, . 2020. Inborn errors of type I IFN immunity in patients with life-threatening COVID-19. Science. 370:eabd4570. 10.1126/science.abd4570 32972995 PMC7857407

