# Supplementary material for: Heterozygous BTNL8 variants in individuals with multisystem inflammatory syndrome in children (MIS-C)
Source: J Exp Med. 2024 Nov 22;221(12):e20240699. doi: 10.1084/jem.20240699 (PMC11586762; doi:10.1084/jem.20240699)
Supplement: Table S1 — shows the breakdown of external cohorts described and the analyses. [file JEM_20240699_TableS1.docx]

Table S1: Breakdown of external cohorts described and the analyses

| **Cohort name** | **Origin of cohort** | **no. MIS-C** | **no. healthy controls** | **no. febrile controls** | **no. COVID-19** | **Analysis** | **Reference** |
| --- | --- | --- | --- | --- | --- | --- | --- |
| COVIDHGE MIS-C | COVIDHGE | 690 | 0 | 0 | 0 | Gene burden |  |
| COVIDHGE COVID19 | COVIDHGE | 0 | 0 | 0 | 189 | Gene burden |  |
| EUCLIDS | EUCLIDS | 0 | 0 | 502 | 0 | Gene burden |  |
| Whole blood transcriptomics* | DIAMONDS, EUCLIDS, PERFORM | 75 | 134 | 535 | 0 | Transcriptomic | Jackson et al., 2023 |
| QuantiFERON RNAseq* | DIAMONDS | 26 | 10 | 10 | 0 | Transcriptomic | Shankar-Hari et al., 2023 |
| SomaScan* | PreVAIL, DIAMONDS, PERFORM | 79 | 24 | 81 | 0 | Proteomics |  |
| ICR1000 | UK 1958 Birth Cohort | 0 | 1000 | 0 | 0 | Gene burden | Ruark et al., 2015 |
