# Supplementary material for: Heterozygous BTNL8 variants in individuals with multisystem inflammatory syndrome in children (MIS-C)
Source: J Exp Med. 2024 Nov 22;221(12):e20240699. doi: 10.1084/jem.20240699 (PMC11586762; doi:10.1084/jem.20240699)
Supplement: Table S2 — shows rare non-synonymous variants in genes previously implicated in primary immunodeficiencies identified in MIS-C patients. [file JEM_20240699_TableS2.docx]

Table S2: Rare non-synonymous variants in genes previously implicated in primary immunodeficiencies identified in MIS-C patients

|  |  |  |  | |  |  | |  |  |  | **Gene level (IUIS)** | |
| --- | --- | --- | --- | --- | --- | --- | --- | --- | --- | --- | --- | --- |
| **Patient** | Gene | Variant | Zygosity | | ACMG | AF (%) | | Ethnicity specific AF (%) | CADD Score | dbSNP | Inheritance | Associated disease |
|  |  | ***COMPLEMENT DEFICIENCIES*** | | | | | | | | |  | |
| **P1** | *CD46* | p.A338V | Het | | Pathogenic | | 1.5 | 0.25 | <10 | rs35366573 | AD | Atypical haemolytic uraemic syndrome |
| **P2** | *CD46* | p.A338V | Het | | Pathogenic | | 1.5 | 0.25 | <10 | rs35366573 | AD | Atypical haemolytic uraemic syndrome |
|  |  | ***COMBINED IMMUNODEFICIENCIES*** | | | | | | | | |  | |
| **P3** | *TBX1* | p.K103del | Het | | Pathogenic | 0.04 | | 0.715 | 20.9 | rs369050575 | AD | Thymic Defects and Congenital Anomalies |
|  |  | ***DISEASES OF IMMUNE DYSREGULATION*** | | | | | | | | |  | |
| **P4** | *CASP10* | p.I406L | Het | | Pathogenic | 0.45 | | 0.005 | 12.49 | rs80358239 | AD | Autoimmune Lymphoproliferative Syndrome |
| **P5** | *PRF1* | p.L17fs*34 | Het | | Pathogenic | 0 | | 0 | 7.814 | - | AR | Familial Hemophagocytic Lymphohistiocytosis |
| **P5** | *PRF1* | p.N252S | Het | | Benign | 0.51 | | 0.969 | 14.37 |  | AR | Familial Hemophagocytic Lymphohistiocytosis |
|  |  | ***BONE MARROW FAILURE*** | | | | | | | | |  |  |
| **P6** | *FANCC* | p.E163fs*30 | | Het | Pathogenic | 0 | | 0 | 33 | rs730881708 | AR | Fanconi Anemia |
| **P6** | *FANCC* | p.H120fs*24 | | Het | Likely Pathogenic | 0 | | 0 | 24.3 | - | AR | Fanconi Anemia |
| **P6** | *FANCC* | p.S119fs*24 | | Het | Likely Pathogenic | 0 | | 0 | 32 | . | AR | Fanconi Anemia |
