# Supplementary material for: Heterozygous BTNL8 variants in individuals with multisystem inflammatory syndrome in children (MIS-C)
Source: J Exp Med. 2024 Nov 22;221(12):e20240699. doi: 10.1084/jem.20240699 (PMC11586762; doi:10.1084/jem.20240699)
Supplement: Table S3 — shows genetic variants identified in MIS-C cohort in OAS-RNAseL pathway. [file JEM_20240699_TableS3.docx]

Table S3: Genetic variants identified in MIS-C cohort in OAS-RNAseL pathway

| **Patient** | **Gene symbol** | **Variant** | **Genotype** | **Computed classificiation** | **CADD score** | **dbSNP (rs)** | **GnomAD Freq (%)** |
| --- | --- | --- | --- | --- | --- | --- | --- |
| P7 | *OAS1* | p.R27C | Het | VUS | 16.11 | 117835961 | 0.001 |
| P8 | *OAS1* | p.D75A | Het | VUS | 23.9 | 148499944 | 0.014 |
| P9, P10, P11, P12 | *OAS1* | p.Q91E | Het | Likely Benign | 19.16 | 150636851 | 0.115 |
| P13, P14 | *OAS1* | p.V135I | Het | VUS | 11.45 | 111902215 | 0.007 |
| P15 | *OAS1* | p.P301L | Het | VUS | 24.8 | 201971047 | 0.01 |
| P16 | *OAS1* | p.G375E | Het | VUS | < 10 | 201042567 | 0 |
| P17 | *OAS2* | p.T133I | Het | VUS | < 10 | 372375150 | 0.002 |
| P18 | *OAS2* | p.R393Q | Het | VUS | 11.28 | 150642424 | 0.091 |
| P19 | *OAS2* | p.G406fs*2 | Het | VUS | 22.9 | - | 0.024 |
| P20 | *OAS2* | p.R535Q | Het | VUS | 13.58 | 200885734 | 0.029 |
| P21 | *OAS2* | p.T687K | Het | VUS | 19.28 | - | 0 |
| P22 | *OAS3* | p.T134I | Het | VUS | < 10 | 200010016 | 0.024 |
| P23 | *OAS3* | p.E179Q | Het | VUS | 22.7 | 45585037 | 0.12 |
| P24 | *OAS3* | p.H264Q | Het | Likely Benign | < 10 | 200894876 | 0.1 |
| P25 | *OAS3* | p.W269X | Het | VUS | 39 | 368028980 | 0.002 |
| P26 | *OAS3* | p.A321T | Het | VUS | < 10 | 749442922 | 0.009 |
| P27 | *OAS3* | p.R331K | Het | VUS | < 10 | 753286423 | 0.006 |
| P28 | *OAS3* | p.E447K | Het | VUS | 15.04 | - | 0 |
| P29 | *OAS3* | p.Q653R | Het | VUS | 11.18 | - | 0 |
| P30 | *OAS3* | p.A920V | Het | VUS | 16.08 | 539747098 | 0.188 |
| P31 | *OAS3* | p.R1053C | Het | VUS | 23.4 | 201242016 | 0.006 |
| P32 | *RNASEL* | p.P62S | Het | VUS | 19.49 | 114166108 | 0.007 |
| P33, P34 | *RNASEL* | p.I97L | Het | Likely Benign | 22.6 | 56250729 | 0.654 |
| P2 | *RNASEL* | p.I101T | Het | VUS | 22.3 | 115634589 | 0.026 |
| P35 | *RNASEL* | p.K146Q | Het | Likely Benign | < 10 | 530816616 | 0.001 |
| P36 | *RNASEL* | p.E265* | Het | VUS | 33 | 74315364 | 0.356 |
| P37 | *RNASEL* | p.K635R | Het | VUS | 26.9 | 781464201 | 0.002 |
