# Supplementary material for: Heterozygous BTNL8 variants in individuals with multisystem inflammatory syndrome in children (MIS-C)
Source: J Exp Med. 2024 Nov 22;221(12):e20240699. doi: 10.1084/jem.20240699 (PMC11586762; doi:10.1084/jem.20240699)
Supplement: Table S4 — shows genes significantly enriched in combined gene burden testing. [file JEM_20240699_TableS4.docx]

Table S4: Genes significantly enriched in combined gene burden testing

| **Gene** | **P-value** | | | | | **Variant Count** | | | | |
| --- | --- | --- | --- | --- | --- | --- | --- | --- | --- | --- |
|  | **EUR** | **AFR** | **AMR** | **SAS** | **COMBINED** | **EUR** | **AFR** | **AMR** | **SAS** | **COMBINED** |
| BTNL8 | <1.00E-06 | 5.48E-04 | 2.00E-06 | 4.63E-03 | <1.00E-06 | 8 | 4 | 6 | 2 | 20 |
| AHNAK2 | 1.66E-01 | 1.06E-03 | 8.00E-06 | 1.43E-01 | <1.00E-06 | 6 | 16 | 21 | 3 | 46 |
| CCDC116 | 2.61E-02 | 4.85E-03 | 5.74E-03 | 9.94E-02 | <1.00E-06 | 2 | 3 | 3 | 1 | 9 |
| FAM209B | <1.00E-06 | 1.00E+00 | 7.89E-02 | 1.00E+00 | <1.00E-06 | 9 | 0 | 3 | 0 | 12 |
| GPR123 | 9.80E-05 | <1.00E-06 | 5.30E-04 | 1.00E+00 | <1.00E-06 | 4 | 8 | 5 | 0 | 17 |
| METRN | 1.00E+00 | <1.00E-06 | 1.00E+00 | 3.35E-02 | <1.00E-06 | 0 | 6 | 0 | 1 | 7 |
| MKI67 | 6.92E-02 | <1.00E-06 | 3.27E-03 | 2.28E-01 | <1.00E-06 | 2 | 8 | 5 | 1 | 16 |
| PRKRA | 1.26E-01 | 1.19E-01 | <1.00E-06 | 6.20E-03 | <1.00E-06 | 1 | 1 | 7 | 2 | 11 |
| RP11-1021N1.1 | <1.00E-06 | 1.00E+00 | 1.00E+00 | 1.00E+00 | <1.00E-06 | 3 | 0 | 0 | 0 | 3 |
| C21orf67 | 4.75E-03 | 1.00E+00 | 3.40E-03 | <1.00E-06 | 2.00E-06 | 1 | 0 | 1 | 1 | 3 |
| COX8C | 3.63E-03 | 1.00E+00 | <1.00E-06 | 1.00E+00 | 2.00E-06 | 1 | 0 | 1 | 0 | 2 |
| ZNF726 | 2.05E-02 | 6.10E-03 | 4.24E-03 | 1.00E+00 | 4.00E-06 | 2 | 3 | 3 | 0 | 8 |
| CLLU1 | 3.70E-03 | 2.73E-02 | <1.00E-06 | 1.00E+00 | 1.40E-05 | 1 | 1 | 1 | 0 | 3 |
| PRAMEF12 | 9.50E-04 | 9.20E-02 | 1.18E-01 | 2.98E-02 | 1.60E-05 | 2 | 1 | 1 | 1 | 5 |
| APOL1 | 1.00E+00 | 8.28E-02 | 6.00E-06 | 1.00E+00 | 2.20E-05 | 0 | 1 | 3 | 0 | 4 |
| TAS2R5 | 1.00E+00 | 6.65E-02 | 8.00E-06 | 1.00E+00 | 2.60E-05 | 0 | 1 | 3 | 0 | 4 |
| INO80C | 2.93E-01 | 2.32E-02 | 8.60E-05 | 1.00E+00 | 3.00E-05 | 1 | 2 | 6 | 0 | 9 |
| OR12D1 | <1.00E-06 | 1.00E+00 | 1.00E+00 | 1.00E+00 | 3.40E-05 | 4 | 0 | 0 | 0 | 4 |
| PMF1-BGLAP | 1.00E+00 | 3.65E-03 | 2.30E-03 | 6.26E-02 | 4.60E-05 | 0 | 2 | 2 | 1 | 5 |
| TRAV6 | 4.02E-04 | 1.00E+00 | 1.36E-01 | 3.81E-02 | 5.40E-05 | 2 | 0 | 1 | 1 | 4 |
| DKFZP779J2370 | 1.00E+00 | 4.95E-03 | 5.02E-04 | 1.00E+00 | 6.20E-05 | 0 | 2 | 2 | 0 | 4 |
| PRR22 | 5.94E-02 | 3.83E-02 | 1.97E-02 | 7.34E-02 | 6.80E-05 | 1 | 2 | 2 | 1 | 6 |
| ARHGEF26 | 1.00E+00 | 1.00E-05 | 6.32E-01 | 4.34E-03 | 7.80E-05 | 0 | 7 | 1 | 4 | 12 |
| FDXACB1 | 1.00E+00 | 2.00E-05 | 1.10E-01 | 2.29E-01 | 8.20E-05 | 0 | 7 | 3 | 1 | 11 |
| DUXA | 1.40E-05 | 1.03E-01 | 1.00E+00 | 1.00E+00 | 1.22E-04 | 3 | 1 | 0 | 0 | 4 |
| TMEM120B | 7.20E-05 | 1.00E+00 | 5.23E-03 | 1.00E+00 | 1.22E-04 | 3 | 0 | 2 | 0 | 5 |
| SNRK | 1.00E+00 | 3.08E-02 | 7.74E-02 | 2.12E-03 | 1.26E-04 | 0 | 2 | 2 | 3 | 7 |
| ADAMDEC1 | 1.00E+00 | 4.95E-02 | 1.53E-02 | 3.60E-05 | 1.30E-04 | 0 | 2 | 3 | 3 | 8 |
| F10 | 3.50E-03 | 1.00E+00 | 1.20E-02 | 5.82E-02 | 1.36E-04 | 2 | 0 | 2 | 1 | 5 |
| SOGA3 | 2.02E-01 | 2.67E-02 | 1.02E-03 | 1.00E+00 | 1.36E-04 | 1 | 2 | 4 | 0 | 7 |
| FOXI2 | 1.00E+00 | 2.73E-02 | 1.66E-04 | 1.00E+00 | 2.04E-04 | 0 | 2 | 3 | 0 | 5 |
| PLA2G2A | 1.00E+00 | 3.60E-05 | 1.00E+00 | 1.00E+00 | 2.16E-04 | 0 | 3 | 0 | 0 | 3 |
| SPON2 | 1.00E+00 | 4.27E-01 | 2.60E-02 | 2.00E-06 | 2.88E-04 | 0 | 1 | 2 | 4 | 7 |
| AKNA | 1.42E-02 | 3.39E-02 | 1.20E-02 | 1.00E+00 | 2.98E-04 | 3 | 3 | 4 | 0 | 10 |
| KLHL21 | 4.82E-03 | 1.18E-02 | 1.18E-01 | 1.00E+00 | 3.48E-04 | 3 | 5 | 2 | 0 | 10 |
| CD164 | 1.27E-01 | 5.62E-03 | 5.77E-02 | 1.00E+00 | 3.70E-04 | 1 | 2 | 1 | 0 | 4 |
| TEX37 | 6.05E-02 | 1.92E-02 | 3.14E-02 | 1.00E+00 | 3.98E-04 | 1 | 3 | 2 | 0 | 6 |
| CENPF | 1.00E+00 | 2.48E-02 | 2.86E-04 | 4.30E-01 | 4.24E-04 | 0 | 6 | 8 | 1 | 15 |
| RP11-216L13.17 | 8.50E-03 | 8.45E-03 | 1.00E+00 | 1.00E+00 | 4.66E-04 | 1 | 1 | 0 | 0 | 2 |
| HLA-C | 2.29E-02 | 5.38E-01 | 2.98E-04 | 1.00E+00 | 4.92E-04 | 5 | 1 | 7 | 0 | 13 |
| LINC01101 | 1.00E+00 | 4.43E-02 | 3.64E-04 | 1.00E+00 | 5.06E-04 | 0 | 2 | 2 | 0 | 4 |
| TRAV13-1 | 6.16E-02 | 1.00E+00 | 2.60E-04 | 1.00E+00 | 5.24E-04 | 1 | 0 | 2 | 0 | 3 |
| BDKRB1 | 8.86E-04 | 1.00E+00 | 1.07E-02 | 1.00E+00 | 5.36E-04 | 4 | 0 | 3 | 0 | 7 |
| KRT84 | 2.07E-01 | 2.64E-01 | 1.40E-02 | 9.96E-03 | 5.46E-04 | 3 | 2 | 7 | 3 | 15 |
| FHL2 | 1.00E+00 | 9.22E-04 | 6.86E-02 | 1.33E-01 | 6.96E-04 | 0 | 3 | 2 | 1 | 6 |
| PEG3 | 6.65E-01 | 5.74E-03 | 9.97E-03 | 3.36E-01 | 7.16E-04 | 1 | 6 | 4 | 1 | 12 |
| DZIP1L | 2.10E-01 | 5.04E-01 | 3.78E-04 | 1.70E-01 | 7.26E-04 | 1 | 1 | 4 | 1 | 7 |
| COPS7A | 1.00E+00 | 9.50E-03 | 4.14E-03 | 1.00E+00 | 7.36E-04 | 0 | 3 | 2 | 0 | 5 |
| PRC1 | 1.00E+00 | 2.76E-01 | 5.28E-04 | 1.40E-01 | 7.36E-04 | 0 | 1 | 4 | 1 | 6 |
| IGLV1-36 | 1.19E-02 | 1.33E-02 | 1.00E+00 | 1.00E+00 | 7.42E-04 | 1 | 1 | 0 | 0 | 2 |
| RNASE3 | 1.06E-03 | 7.94E-02 | 1.00E+00 | 1.00E+00 | 8.02E-04 | 2 | 1 | 0 | 0 | 3 |
| CABS1 | 5.01E-02 | 1.00E+00 | 4.74E-04 | 1.00E+00 | 8.58E-04 | 1 | 0 | 2 | 0 | 3 |
| RNF149 | 8.00E-02 | 5.56E-02 | 2.33E-01 | 5.74E-02 | 8.80E-04 | 2 | 2 | 1 | 2 | 7 |
| CTD-2215E18.1 | 1.00E+00 | 1.00E+00 | 1.20E-05 | 1.00E+00 | 9.24E-04 | 0 | 0 | 2 | 0 | 2 |
| SKA3 | 6.00E-05 | 4.68E-01 | 8.38E-02 | 2.85E-01 | 9.26E-04 | 3 | 1 | 1 | 1 | 6 |
| SLC9A3 | 2.95E-01 | 1.00E+00 | 1.00E+00 | 6.00E-06 | 9.30E-04 | 1 | 0 | 0 | 6 | 7 |
