# Supplementary material for: Heterozygous BTNL8 variants in individuals with multisystem inflammatory syndrome in children (MIS-C)
Source: J Exp Med. 2024 Nov 22;221(12):e20240699. doi: 10.1084/jem.20240699 (PMC11586762; doi:10.1084/jem.20240699)
Supplement: Table S5 — shows COVID-19 Host Genetics Initiative meta-analysis gene burden results for BTNL8 (adapted from supplementary material of Butler-Laporte et al. (2022). [file JEM_20240699_TableS5.docx]

Table S5: COVID-19 Host Genetics Initiative (HGI) meta-analysis gene burden results for BTNL8 (adapted from supplementary material of (Butler-Laporte et al., 2022)

| **Gene** | **COVID-19** | **Mask** | **MAF** | **Meta-analysis** | | | **N Cases 0\|1\|2** |  | **N Controls 0\|1\|2** |
| --- | --- | --- | --- | --- | --- | --- | --- | --- | --- |
|  | **Phenotype** |  | **Filters** | **Effect** | **StdErr** | **Pvalue** | **Burden Test** |  | **Burden Test** |
| BTNL8 | Susceptibility | M4* | <1% | -0.058 | 0.066 | 0.376 | 27793\|225\|9 |  | 596112\|4896\|235 |
| BTNL8 | Hospitalisation | M4* | <1% | 0.024 | 0.117 | 0.84 | 12015\|92\|5 |  | 587986\|4704\|201 |
| BTNL8 | Severity | M4* | <1% | 0.045 | 0.279 | 0.871 | 4958\|32\|2 |  | 570549\|4620\|197 |

*M4 mask: pLOF and missense variants predicted to be deleterious in silico as described in Butler-Laporte et al., 2022
