# Supplementary material for: Heterozygous BTNL8 variants in individuals with multisystem inflammatory syndrome in children (MIS-C)
Source: J Exp Med. 2024 Nov 22;221(12):e20240699. doi: 10.1084/jem.20240699 (PMC11586762; doi:10.1084/jem.20240699)
Supplement: Table S6 — shows CNV frequency in MIS-C cohort compared to gnomAD. [file JEM_20240699_TableS6.docx]

Table S6: CNV frequency in MIS-C cohort compared to gnomAD

|  | **Genotype Frequency** | | | **Allele Frequency** |
| --- | --- | --- | --- | --- |
|  | 0/0 | 0/DEL | DEL/DEL | DEL |
| Observed  in MIS-C | 63.20% | 29.40% | 7.40% | 22.10% |
| Expected  (gnomAD) | 62.70% | 31.80% | 5.60% | 21.40% |
