# Supplementary material for: Heterozygous BTNL8 variants in individuals with multisystem inflammatory syndrome in children (MIS-C)
Source: J Exp Med. 2024 Nov 22;221(12):e20240699. doi: 10.1084/jem.20240699 (PMC11586762; doi:10.1084/jem.20240699)
Supplement: Table S7 — shows rare variant burden analysis at the protein domain level. [file JEM_20240699_TableS7.docx]

Table S7: Rare variant burden analysis at the protein domain level

| **Gene** | **Domain**  **(PFAM)** | **Cohort**  **Allele Count** | **Pvalue** |
| --- | --- | --- | --- |
| BTNL8 | PF13765/PF00622 | 5 | 1.40E-05 |
| AKNA | PF12443 | 11 | 1.60E-05 |
| KLHL21 | PF00651 | 6 | 5.60E-05 |
| KLHL21 | PF00651 | 6 | 9.00E-05 |
| PLA2G2A | PF00068 | 3 | 1.19E-03 |
| ARHGEF26 | PF00169 | 6 | 1.28E-03 |
| FHL2 | PF00412 | 4 | 2.05E-03 |
| SPON2 | PF06468 | 7 | 3.47E-03 |
| F10 | PF14670 | 4 | 8.32E-03 |
| F10 | PF00089 | 4 | 9.10E-03 |
| ZNF726 | PF00096 | 4 | 1.25E-02 |
| CD164 | PF05283 | 3 | 1.52E-02 |
| FDXACB1 | PF03147 | 3 | 1.77E-02 |
| KRT84 | PF00038 | 8 | 2.20E-02 |
| BDKRB1 | PF00001 | 8 | 2.22E-02 |
| CCDC116 | PF15774 | 9 | 2.36E-02 |
| GPR123 | PF00002 | 4 | 2.48E-02 |
| RNF149 | PF02225 | 3 | 2.82E-02 |
| CENPF | PF10473 | 2 | 3.37E-02 |
| SLC9A3 | PF00999 | 3 | 3.93E-02 |
| FOXI2 | PF00250 | 2 | 5.43E-02 |
| COPS7A | PF18392 | 2 | 5.69E-02 |
| FDXACB1 | PF10354 | 5 | 1.13E-01 |
| ADAMDEC1 | PF00200 | 2 | 1.16E-01 |
| RNASE3 | PF00074 | 3 | 1.24E-01 |
| CENPF | PF10481 | 3 | 1.25E-01 |
| KRT84 | PF16208 | 5 | 1.37E-01 |
| PRR22 | PF15776 | 6 | 1.80E-01 |
| PRC1 | PF03999 | 3 | 2.03E-01 |
| BTNL8 | PF008205 | 2 | 2.18E-01 |
