# Supplementary material for: Heterozygous BTNL8 variants in individuals with multisystem inflammatory syndrome in children (MIS-C)
Source: J Exp Med. 2024 Nov 22;221(12):e20240699. doi: 10.1084/jem.20240699 (PMC11586762; doi:10.1084/jem.20240699)
Supplement: Table S8 — shows domain-level BTNL8 rare variant burden in COVID-HGE cohort. [file JEM_20240699_TableS8.docx]

Table S8: Domain-level BTNL8 rare variant burden in COVID-HGE cohort

| **BTNL8** | **PFAM** | **Allele Count** | | **Burden** |
| --- | --- | --- | --- | --- |
| **domain** | **identifier** | **COVID-HGE** | **ICR1000** | **Permutation** |
|  |  | **(n=300)** | **(n=1000)** | **P-value** |
| IgV | PF07686 | 7 | 26 | 5.05E-01 |
| IgC | PF008205 | 4 | 5 | 3.46E-02 |
| B30.2 | PF13765/PF00622 | 8 | 7 | 7.00E-04 |
